# Lewy Bodies Are Not Associated With Neuronal or Synaptic Loss in Dementia With Lewy Bodies

**DOI:** 10.1111/nan.70085

**Published:** 2026-06-10

**Authors:** Jade I. Hawksworth, Eddie Kirkby‐Geddes, Searlait Thom, Joe O'Neill, Amelia Ikwue, Lucy Wood, Tiago F. Outeiro, Daniel Erskine

**Affiliations:** ^1^ Translational and Clinical Research Institute Newcastle University, Campus for Ageing and Vitality Newcastle Upon Tyne UK; ^2^ Department of Experimental Neurodegeneration, Center for Biostructural Imaging of Neurodegeneration University Medical Center Göttingen Göttingen Germany; ^3^ Faculdade de Medicina e Ciências Biomédicas, Algarve Biomedical Center Research Institute (ABC‐Ri) Universidade do Algarve Faro Portugal; ^4^ Scientific Employee With an Honorary Contract at Deutsches Zentrum für Neurodegenerative Erkrankungen (DZNE) Göttingen Germany

**Keywords:** alpha‐synuclein, dementia with Lewy bodies, histopathology, neuronal density, synaptic proteins

## Abstract

**Aims:**

The misfolding and accumulation of the protein *α*‐synuclein (*α*Syn) into cytoplasmic inclusions termed Lewy bodies (LBs) and Lewy neurites is the defining neuropathological feature of LB diseases, such as Parkinson's disease (PD) and dementia with Lewy bodies (DLB). The loss of neurons and/or synapses has been postulated to underlie the clinical syndrome of DLB. The present study sought to elucidate the relationship between LB burden and neuronal and synaptic loss in DLB.

**Methods:**

*Post‐mortem* brain tissue from the cingulate gyrus and inferior temporal gyrus, two regions vulnerable to LB pathology, was obtained from DLB (*N* = 20) and control cases (*N* = 20). Formalin‐fixed paraffin‐embedded tissue was stained to quantify LB, Alzheimer‐type pathology and a neuronal marker. Frozen tissue from the contralateral hemisphere was processed for immunoblotting to compare the abundance of synaptic markers across cases.

**Results:**

Across both regions, no evidence of reduced total neuronal density was observed, but a modest reduction in parvalbumin interneurons was observed in the cingulate gyrus, and there were only modest reductions in some synaptic markers in DLB. LB burden was markedly variable across DLB cases but was not associated with any synaptic marker abundance or neuronal density.

**Conclusions:**

Taken together, these findings do not support an association between LB density and neuronal or synaptic loss in DLB, even in regions with particularly high burdens of LBs, such as the cingulate gyrus. These findings suggest that the link between *α*Syn proteinopathy and disease requires further investigation.

## Summary

- Quantification of Lewy body (LB) density in the cingulate gyrus and inferior temporal gyrus indicated high variability across DLB cases.
- LB density was associated with amyloid‐β abundance.
- Total neuronal density was not different between groups; however, parvalbumin interneurons were significantly reduced in the DLB cingulate gyrus.
- Synaptic markers were only modestly lower in DLB tissue compared to controls.
- LB density, with either a pan‐*α*Syn or *α*Syn pS129 antibody, was not associated with neuronal density or synaptic loss, even in regions with particularly high burdens of pathology, such as the cingulate gyrus.

## Graphical abstract

Lewy bodies are thought to be central drivers of neurodegeneration and synaptic loss in dementia with Lewy bodies (DLB). In this article, Hawksworth, Kirkby‐Geddes et al. observe no associations between Lewy body density and loss of neurons or synaptic markers in DLB. These findings challenge the assumption that Lewy body formation is a driver of neurodegeneration in DLB.

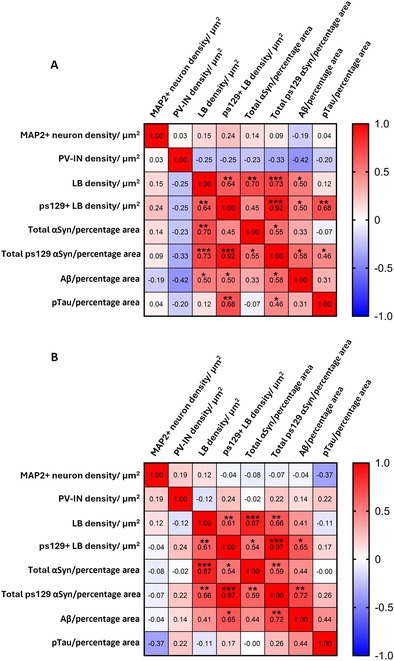

**Abbreviations**
*α*Syn: alpha‐synuclein
CG: cingulate gyrus
DLB: dementia with Lewy bodies
ITG: inferior temporal gyrus
LB: Lewy body
NHS: National Health Service
PD: Parkinson's disease
PSD‐95: Postsynaptic Density Protein 95

## 1 Introduction

Dementia with Lewy bodies (DLB) is a common neurodegenerative dementia disorder characterised by the core clinical features of cognitive fluctuations, visual hallucinations, parkinsonism and REM sleep behaviour disorder [1]. Treatment options for DLB are presently limited to targeting specific symptoms due to a lack of disease‐modifying therapies [2]. DLB has a progressive course associated with a particularly high burden to caregivers, meaning the development of disease‐modifying therapies is a pressing priority [3]. However, the development of such therapies has been impeded, to some extent, by an incomplete understanding of the aetiology of DLB.

The defining neuropathological feature of DLB is the presence of spherical intraneuronal inclusion bodies of the protein *α*Syn termed Lewy bodies (LBs) [1]. Notably, LBs are also the defining feature of the related condition, Parkinson's disease (PD) [4]. LB pathology progresses in a stereotyped manner in most cases and is thought to spread from the dorsal motor nucleus of the vagus nerve in the medulla through the midbrain, limbic and cortical regions [5]. The topographical progression of LB pathology is thought to mirror the progression of symptoms in PD, where it presents as a motor and autonomic disorder that ultimately progresses to cognitive impairment in many cases. In addition to LB pathology, the brains of individuals with DLB frequently manifest Alzheimer‐type co‐pathology of amyloid‐*β* and hyper‐phosphorylated tau, which may underlie a more rapid decline in DLB [6, 7, 8].

LBs have long been thought to be a key contributor to the disease process of PD and DLB primarily due to their association with disease states and their presence in regions vulnerable to neuronal loss, though it remains unclear whether they drive, or are useful markers of, the neurodegenerative process [4, 9]. Previous studies have reported that LBs are rich in organelles, and studies that have attempted to model this process in vitro have reported that sequestration of organelles is detrimental to cellular health and leads to neuronal and synaptic loss [10, 11]. In contrast, previous studies using post‐mortem brain tissue from PD patients have reported that LBs are not associated with cell death markers, neuronal loss precedes the formation of LBs in some regions, and neuronal loss occurs in regions without LBs and vice versa [12, 13, 14, 15]. Taken together, there is continued controversy about whether LBs underlie the neurodegenerative changes associated with disorders such as PD and DLB.

To help address the controversy regarding the role of LBs in neuronal and synaptic loss in DLB, the present study sought to determine whether the abundance of LBs was associated with neuronal density or the abundance of synaptic markers in post‐mortem DLB brain tissue. It follows that if LBs underlie neurodegenerative changes, then their abundance would be associated with the degree of neuronal and synaptic loss.

## 2 Methods

### 2.1 Case Selection

Formalin‐fixed paraffin‐embedded brain tissue, and frozen tissue from the contralateral hemisphere, were obtained from DLB (*N* = 20) and control (*N* = 20) cases held at Newcastle Brain Tissue Resource (NBTR). To minimise bias in selection, the 20 most recent DLB cases were obtained at NBTR, which fulfilled our criteria of Lewy Braak stage 6, a clinico‐pathological consensus diagnosis of DLB (Tables 1 and 2 and Figure S1), the absence of other neurodegenerative diseases or significant vascular pathology and the presence of only low or intermediate Alzheimer‐type pathology. Low or intermediate Alzheimer‐type pathology was defined as a Braak stage of IV or less [5, 16]. Controls were included if they had no or minimal LB pathology (Braak stage 1 or less) and the absence of other significant neurodegenerative diseases, including a Braak stage of III or less, and matched where possible to DLB cases for age and sex. Ethical approval was granted by the NBTR ethical review board (REF: 24/NE/0012, Newcastle and North Tyneside 1 National Health Service [NHS] Research Ethics Committee).

**TABLE 1:** Disease duration and presentation. The years from diagnosis until death are included as disease duration; the presence or absence of core symptoms of DLB, as outlined in McKeith criteria, is included for each case [1].

Participant	Age at onset (years)	Disease duration (years)	Initial symptoms	Visual hallucinations (+/−)	Cognitive fluctuations (+/−)	REM sleep behaviour disorder (+/−)	Parkinsonism (+/−)
DLB 1	78	1	Falls, depression, parkinsonism	Yes	No	Possible	Yes
DLB 2^a^	84	9	Parkinsonism	Yes	Yes	Possible	Yes
DLB 3	77	4	Decline in cognitive function, episodic disorientation, visual hallucinations	Yes	No	Yes	Yes
DLB 4	52	17	Obsessional, hallucinations, decline in executive function	Yes	Yes	None noted	Yes
DLB 5	72	8	Anxiety, phobia, cognitive impairment	None noted	None noted	None noted	Yes
DLB 6	69	8	Fatigue, shuffling, falls, hypersalivation, visual disturbances, hallucinations (people and animals), tremor	Yes	Yes	Yes	Yes
DLB 7	80	1.5	Cognitive impairment, drooling, bilateral parkinsonism	Delusions but no hallucinations	No	No	Yes
DLB 8	70	4	Memory impairment, falls, tremor	Yes	Yes	No	Yes
DLB 9	70	9	RBD, memory impairment, parkinsonism	Yes	Yes	Yes	Yes
DLB 10	73	8	No information	No information	No information	No information	No information
DLB 11	71	6	Memory impairment, wordfinding problems, visual hallucinations	Yes	None noted	None noted	None noted
DLB 12	76	4	Visuospatial difficulties, disorientation, recall problems	Yes	Yes	Possible	Yes
DLB 13	74	10	Visual hallucinations, parkinsonism and falls	Yes	Yes	Yes	Yes
DLB 14	60	11	Mild memory and executive function impairment	Yes	Yes	Yes	Yes
DLB 15	66	5	Changes in personality, word finding and organisation	Possible	Yes	None noted	No
DLB 16	77	4	Declining memory and executive function, visual hallucinations (nocturnal), fatigue	Yes	Yes	None noted	None noted
DLB 17	72	6	Memory, spatial awareness and concentration deficits	Yes	Yes	No	Yes
DLB 18	79	2	Mild forgetfulness and disorientation	Yes	Yes	Yes	Possible mild
DLB 19	67	6	Mild memory, concentration and attention difficulties. Long history of depression/anxiety	No	Yes	None noted	Yes
DLB 20	87	4	Depression, visual hallucinations, REM sleep disorder	Yes	No	Yes	No

Abbreviations: RBD = rapid eye movement (REM) sleep behaviour disorder (RBD); REM = rapid eye movement.

^a^ The participant DLB 2 was removed from the dataset once it was established that their primary presenting symptom was Parkinsonism.

**TABLE 2:** Case demographics. Demographic data for the cases included in this study are outlined. Braak Lewy refers to the LB staging scheme outlined by Braak and colleagues [5]. Braak tau refers to the staging scheme of neurofibrillary tau pathology outlined by Braak and colleagues [16]. Thal amyloid refers to the phase of amyloid‐*β* deposition, as outlined by Thal and colleagues [17]. CERAD refers to neuritic plaque pathology grading, as outlined by Mirra and colleagues [18].

Participant ID	Age	Sex	Braak Lewy	Braak Tau	Thal amyloid	CERAD
Control 1	78	M	0	III	3	Sparse
Control 2	80	F	0	III	2	Negative
Control 3	66	F	0	I	0	Negative
Control 4	92	F	0	III	5	Moderate
Control 5	75	F	0	II	0	Negative
Control 6	76	F	0	II	1	Negative
Control 7	84	M	1	II	1	Negative
Control 8	86	M	0	III	4	Negative
Control 9	85	F	0	III	2	Negative
Control 10	81	M	0	III	1	Negative
Control 11	91	M	0	II	3	Negative
Control 12	73	M	0	III	2	Negative
Control 13	71	F	0	I	2	Negative
Control 14	63	M	0	I	3	Negative
Control 15	82	M	0	II	0	Negative
Control 16	81	F	0	I	3	Negative
Control 17	59	M	0	I	3	Negative
Control 18	69	F	0	I	2	Negative
Control 19	65	M	0	0	2	Negative
Control 20	91	F	0	II	0	Negative
DLB 1	79	F	6	I	3	Negative
DLB 2	93	F	6	IV	5	Moderate
DLB 3	81	M	6	III	4	Sparse
DLB 4	68	M	6	II	4	Sparse
DLB 5	78	M	6	II	2	Negative
DLB 6	77	M	6	III	2	Negative
DLB 7	82	M	6	I	1	Negative
DLB 8	74	M	6	II	3	Negative
DLB 9	79	M	6	II	4	Negative
DLB 10	73	M	6	III	5	Negative
DLB 11	77	M	6	IV	5	High
DLB 12	80	M	6	III	3	Negative
DLB 13	84	M	6	II	3	Negative
DLB 14	73	M	6	III	1	Negative
DLB 15	71	M	6	III	4	Moderate
DLB 16	81	M	6	III	3	Moderate
DLB 17	78	M	6	III	4	Moderate
DLB 18	81	M	6	III	4	Moderate
DLB 19	73	M	6	III	4	Negative
DLB 20	91	F	6	III	4	Moderate

### 2.2 Histological Tissue Preparation and Analysis

Formalin‐fixed paraffin‐embedded tissue blocks from the cingulate gyrus (CG) and inferior temporal gyrus (ITG), regions chosen due to their susceptibility to LB pathology in DLB, were cut into 5‐μm sections and mounted onto glass slides. Sections were dewaxed in HistoClear (National Diagnostics, Atlanta, GA, USA) and rehydrated through a graded series of ethanol solutions to water, prior to antibody‐specific antigen retrieval (Table S1). To quench endogenous peroxidase activity, sections were incubated in 3% hydrogen peroxide prior to washing and incubation in primary antibodies (Table S1). Antibody labelling was visualised with MACH4 universal‐HRP detection system (BioCare Medical, Pacheco, CA, USA), counter‐stained with haematoxylin and mounted with DPX (CellPath, Newtown, Wales).

**FIGURE 1:**
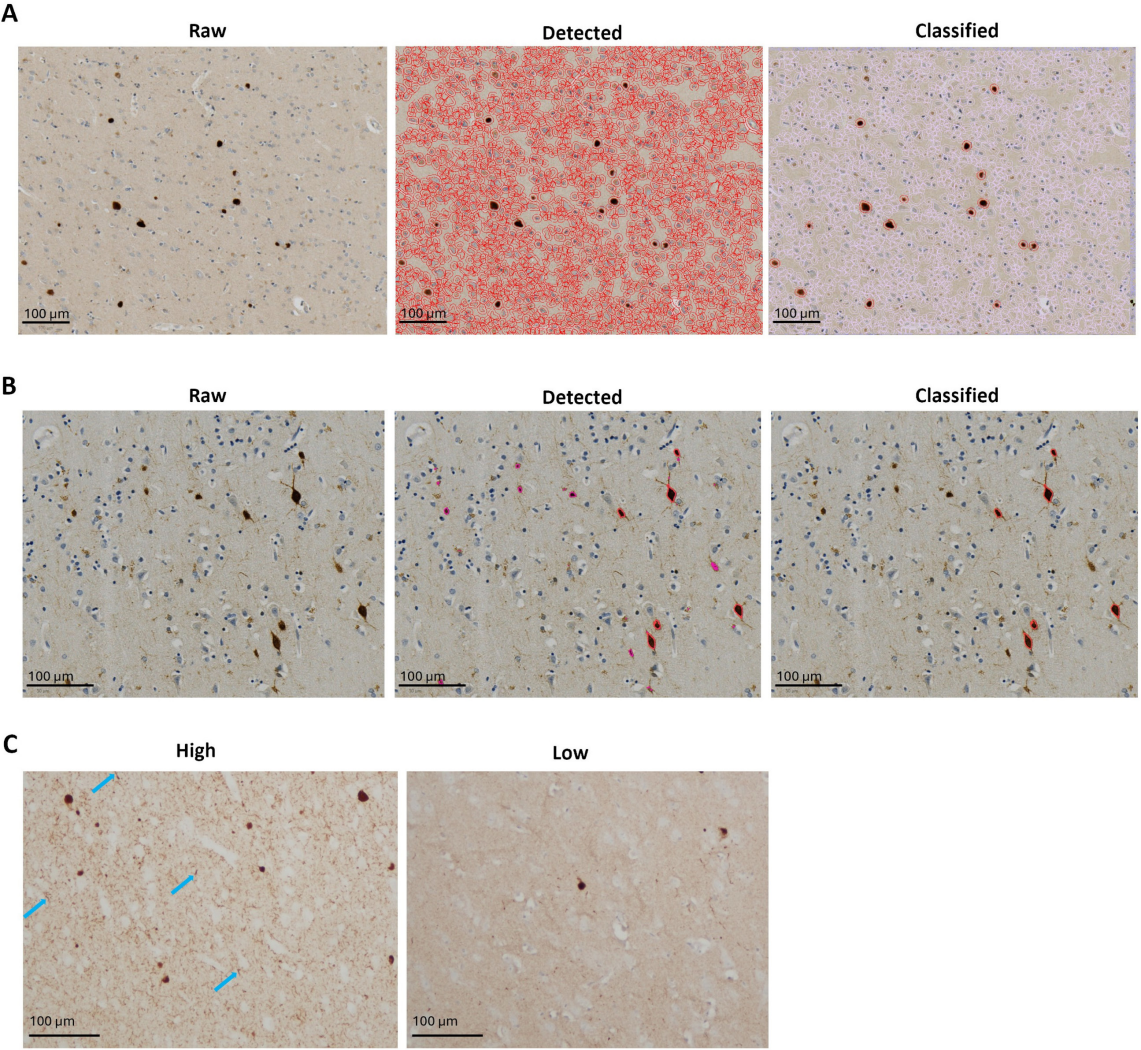
The QuPath detection and classification features can recognise LB on immunohistochemistry slides. (A) Using DLB17 stained for KM51 as an example, a raw image at 20X magnification is shown next to the output of the cell detection (identified objects in red), and the output of the composite classifier, where excluded features are in blue and LB in red. (B) Using DLB17 case stained with parvalbumin as an example, a raw image at 10X magnification is shown next to the output of the cell detection (identified objects in red [classified] and pink [unclassified]), and the output of the classifier, where positive parvalbumin interneurons are shown in red. (C) An example of DLB cases with high and low KM51 LB pathology in the CG at 10X magnification (DLB 1, DLB 5). While only LB are counted using the classifier, dendritic staining (blue arrows) is also captured in the total*α*Syn percentage area.

Stained sections were scanned on an Axioscan‐7 slide scanning system (Zeiss, Oberkochen, Germany) at 10X magnification and imported into QuPath for subsequent analysis [19]. Regions of interest corresponding to the CG and ITG were manually drawn in QuPath, with the coronal face of each gyrus measured in full, from the white matter to the pial surface, and from the depths of both sulci. LB density was optimised on QuPath using a threshold combined with the AI function, under the supervision of an operator (EKG) who checked for correct staining of structures. The cell detection feature was used to identify individual objects based on optical density (Figure S2 and Table S2). Parameters such as background sigma and max area were adjusted to optimise cell detection across all brain sections. After cell detection, a composite classifier was applied, made up of two classifiers, to detect the number of LBs in the annotation. First, LB classification was achieved using the object classification feature, which was trained on a subset of annotations to distinguish LBs from regular cell detections (Figure 1). Finally, the single measurement classifier tool was used to apply a minimum calliper to exclude objects below 10 μm for consistency and to ensure only LBs were included. A manual check of the measurements was performed using the ‘show detection measurements’ feature, and any false positives, such as artefacts, were removed from the count. Once objects had been screened, they were then divided by the annotation area to provide LB density (Tables S3 and S4).

The validity of the composite classifier was tested against a manual count of four DLB cases for the entire annotation. The grid tool was used to systematically ensure the entire region was counted, and the measuring tool was applied to exclude LBs smaller than 10 μm. Agreement between manual and composite classifier counts was assessed using Pearson's correlation coefficient, which showed a strong positive correlation (*r* = 0.999), indicating high consistency.

Determination of neuronal density was performed using sections stained for either the neuronal microtubule marker, MAP 2, or the calcium‐binding protein, parvalbumin. MAP 2 is a microtubule protein enriched in neurons, and is typically employed as a pan‐neuronal marker in studies of human post‐mortem brain tissue [20]. Parvalbumin is a calcium‐binding protein that labels a subset of cortical interneurons characterised by their fast‐spiking activity patterns, rendering them particularly reliant on mitochondrial function and marked susceptibility to degeneration following mitochondrial dysfunction [21]. Variation in staining intensity of neurons labelled by MAP 2 precluded automatic detection using the cell classifier protocol, and attempts to use this did not generate data that passed our quality control checks. As a result, MAP 2+ neurons were manually counted by an operator (EKG) within a manually drawn area in the centre of the gyral crest encompassing all cortical layers from the pial surface to the white matter and typically consisting of an area of 1000 μm^2^. This equated with a mean of 132 neurons per CG and 142 neurons per ITG (Tables S5 and S6). Parvalbumin interneurons were counted in a manually drawn region of interest corresponding with the gyral crest of the gyrus being studied, using the object classifier in QuPath based on an intensity threshold combined with a size exclusion threshold that removed any objects smaller than 45 μm^2^ (Figure 1 and Tables S7–S10).

The percentage area immunoreactive for *α*Syn, p*α*Syn, amyloid‐*β* and tau was evaluated in QuPath using a conventional threshold to detect DAB staining and not background. For amyloid‐*β* immunostaining, a minimum size threshold of 100 μm^2^ was employed to ensure intracellular amyloid‐*β* was not quantified [22]. Due to inter‐case variability in staining intensities, bespoke thresholds were manually applied per case to separate signal from background over a range of background staining intensities (Figure S2). All analyses were performed blind to diagnosis.

### 2.3 Frozen Tissue Preparation and Analysis

Approximately 250 mg of CG and ITG tissue from the contralateral hemisphere of the same cases described in Table 2 were homogenised using a rotator‐stator homogeniser in 10× 0.2M TEAB supplemented with protease inhibitor (cOmplete, Roche, Basel, Switzerland). Total protein per sample was quantified using the Bradford assay, and samples were made up to 1 μg/μL in LDS sample buffer (Invitrogen, Waltham, MA, USA) and sample reducing agent (DTT; Invitrogen, Waltham, MA, USA). The 10‐μg total protein per sample was loaded onto 5%–12% Bis‐Tris Midi protein gels with a 1‐mm pore size and electrophoresed at 200 V, 3000 mA, 350 W for 27 min (synaptophysin, synaptotagmin) or 40 min (gephyrin, PSD‐95).

Samples were transferred to nitrocellulose membranes using an iBlot2 semi‐dry transfer device (Invitrogen, Waltham, MA, USA) at 120V for 9 min, then protein loading was visualised with Ponceau S (Thermo Fisher Scientific, Waltham, MA, USA) staining and imaged on an iBright 1500 imager (Invitrogen, Waltham, MA, USA). After washing, membranes were blocked in 5% bovine serum albumin (Sigma‐Aldrich, St. Louis, MO, USA) for 1 h prior to overnight incubation at 4°C in primary antibody suspended in blocking solution, as outlined in Table S11. We used primary antibodies against two presynaptic markers, synaptophysin and synaptotagmin, and two postsynaptic markers, postsynaptic density protein 95 (PSD‐95) and gephyrin. Synaptophysin, PSD‐95 and gephyrin were previously demonstrated to be significantly reduced in the primary visual cortex in DLB cases [23]. PSD‐95 and gephyrin are scaffolding proteins found in excitatory and inhibitory synapses, respectively, so their inclusion enabled us to investigate whether there are specific alterations to excitatory or inhibitory synapses [24]. Synaptotagmin was included as a further pre‐synaptic marker that has been previously identified as dysregulated in DLB [25].

After primary antibody incubation, membranes were washed and then incubated in an HRP‐conjugated secondary antibody for 1 h at room temperature and visualised on an iBright 1500 imager (Invitrogen, Waltham, MA, USA) using West Femto ECL solution (Thermo Fisher Scientific, Waltham, MA, USA). The abundance of proteins of interest was analysed by measuring the area under the curve of the band corresponding to the canonical protein, as outlined on UniProt [26] and normalised to the total protein as observed with Ponceau from the total lane per sample.

Group‐level differences in synaptic marker abundance were measured in 10 control cases compared to 10 DLB cases per region, as the full cohort could not fit on one western blot gel, and it is not recommended to make comparisons across western blot membranes. The 10 cases and controls used for this analysis were chosen as the first 10 from a list based on year of death. Associations between LB burden and synaptic markers used all 19 DLB cases on each membrane to increase power to detect an association, if one exists. Western blot images were analysed using ImageJ [27].

### 2.4 Statistical Analysis

Statistical analysis was performed using GraphPad Prism (GraphPad Prism 10.1.2, GraphPad Software, Boston, Massachusetts, USA, www.graphpad.com). For group level differences, normality of data and homogeneity of variance were evaluated using Shapiro–Wilk tests and visual inspection of *Q*–*Q* plots and parametric or non‐parametric tests were used, as appropriate. To determine associations between LB density and neuronal density, and measures of synaptic proteins, correlation matrices and multiple linear regressions were performed in Graphpad Prism.

## 3 Results

### 3.1 Neuropathological Characterisation of DLB and Control Cases

**FIGURE 2:**
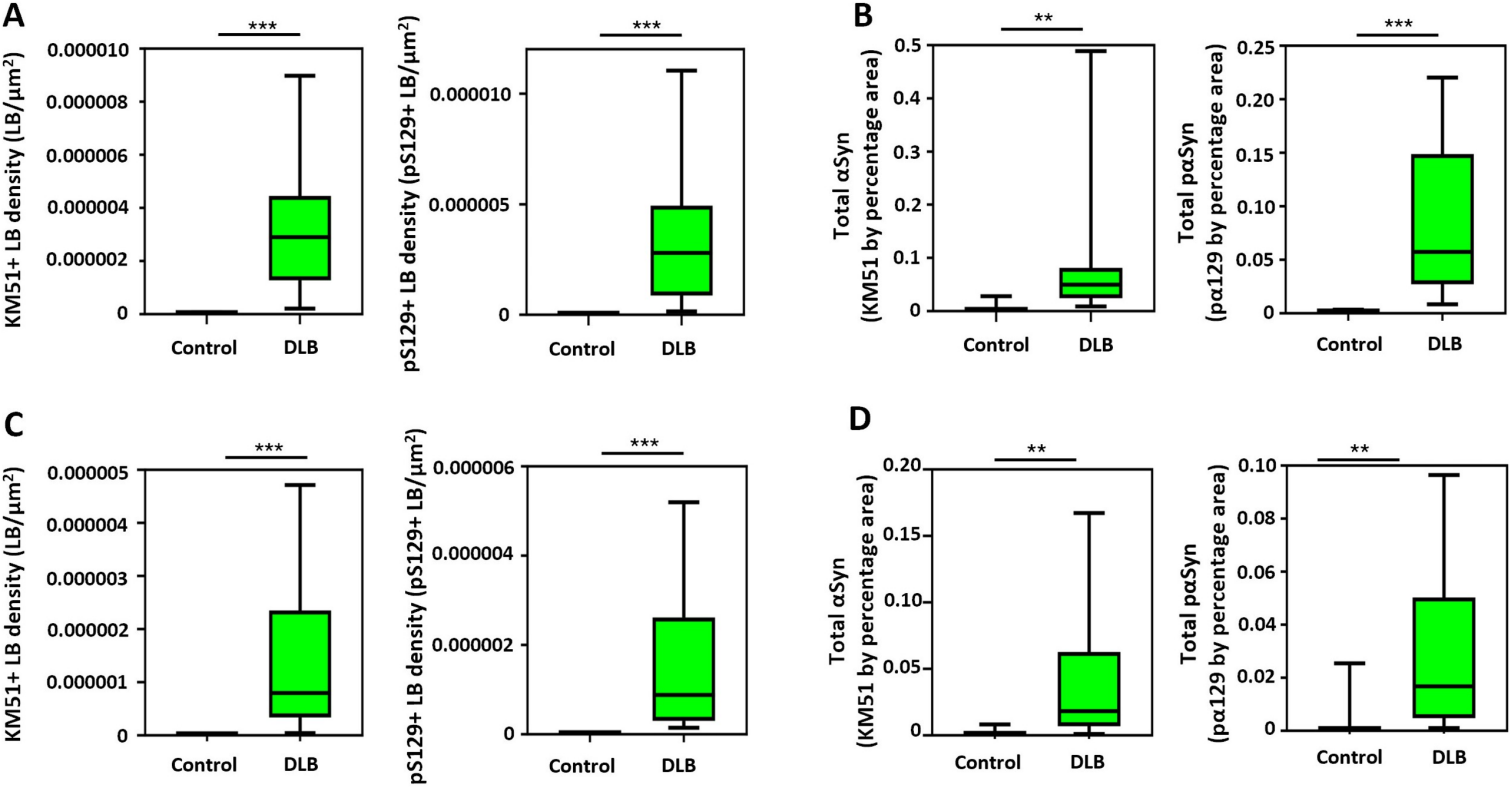
A count of LB density reveals the presence of LB in DLB. LB were quantified as either KM51 + pS129 + inclusions per micrometre (μm), and neurons were counted as MAP 2+ cell bodies per μm. (A) Counts of LBs using KM51+ and pS129+ in the CG. (B) Total *α*Syn and total p*α*Syn measured by percentage area in the CG. C. Counts of LB using KM51+ and pS129+ in the ITG. D. Total *α*Syn and total p*α*Syn measured by percentage area in the CG. *** = *p* value of below 0.001 on a Welch's *t* test.

Evaluation of LB density suggested, unsurprisingly, that LB density was higher in DLB cases compared to controls, in both the CG and the ITG, and irrespective of whether LBs were counted using the KM51 or pS129 antibody (Figure 2).

LB density was highly variable across DLB cases, with the lowest and highest density cases differing by a factor of up to 107× (complete LB density data is available in Tables S3 and S4). LB density was approximately twice as high in the CG compared to the ITG, irrespective of whether it was measured with KM51 (*t* = 2.653, df = 36, *p* = 0.0118) or pS129 (*t* = 2.399, df = 27.28, *p* = 0.0235). There was no significant difference in LB density measured by KM51 compared to pS129 within cases in the CG (*t* = 0.3873, df = 34.04, *p* = 0.7010) or ITG (*t* = 0.3151, df = 35.78, *p* = 0.7545). It was notable that several cases showed striking differences in LB density depending on the marker used; for example, DLB 2 and DLB 16 in the CG, and DLB 11 and DLB 17 in the ITG (Figure S3). These findings suggest the abundance of LBs measured by pS129 is not always a proxy for the abundance measured with a pan‐*α*Syn marker, such as KM51.

**FIGURE 3:**
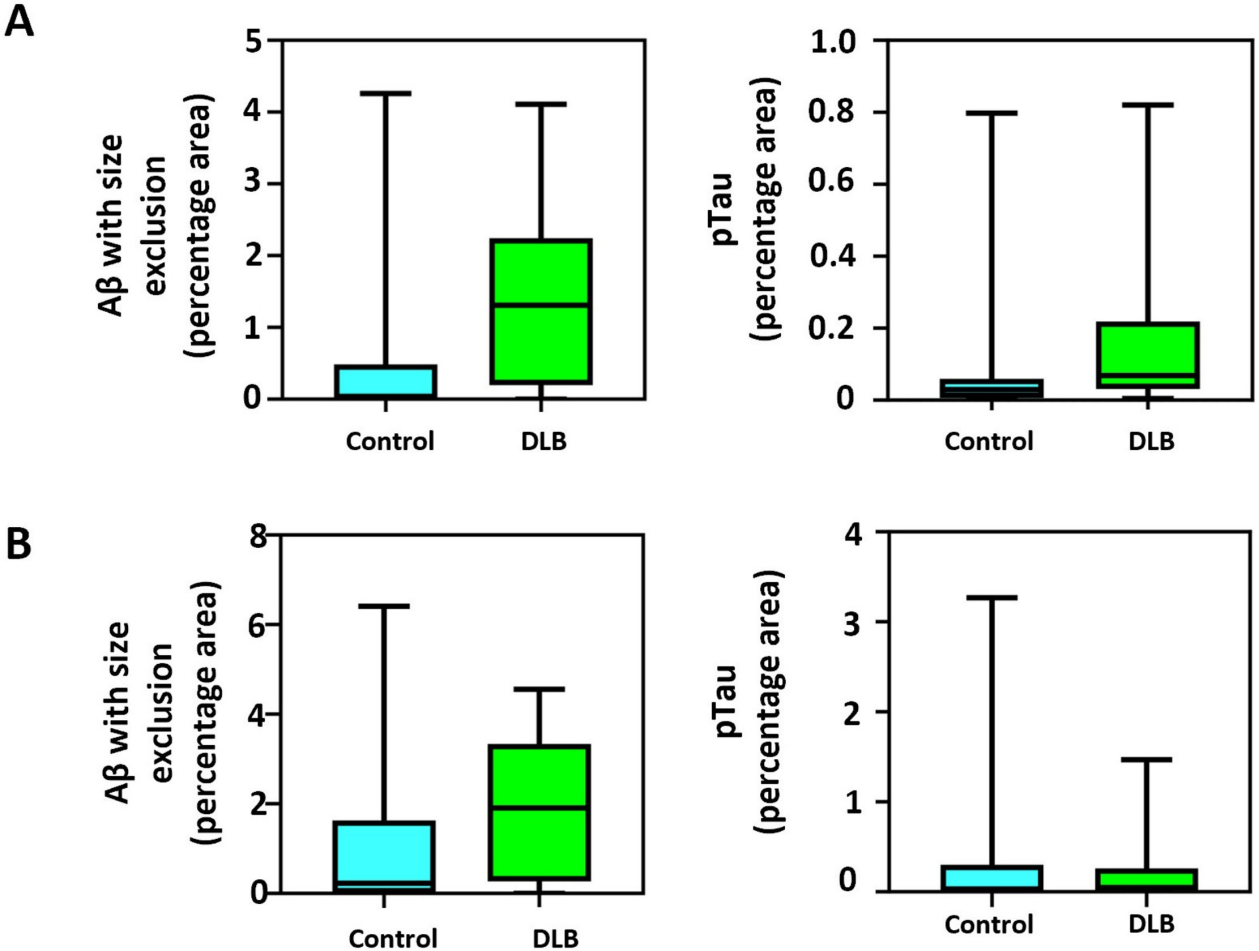
Concomitant Alzheimer‐type pathology is present in DLB. Amyloid‐*β* is presented as a percentage area with a size exclusion removing aggregates smaller than 100 μm^2^, and tau is presented as a percentage area. (A) Amyloid‐*β* and tau pathology in the CG of DLB participants. (B) Amyloid‐*β* and tau pathology in the ITG of DLB participants. ** = *p* value of below 0.01 on a Welch's *t* test.

Total *α*Syn and total p*α*Syn pathology were also quantified in QuPath as percentage area immunoreactivity, using the same sections as used for LB counting (Table 2). *α*Syn pathology is present in DLB brain in the form of LBs, in addition to neurites and smaller aggregates (Figure 1), and, as expected, was significantly more abundant in both the CG (*t* = 3.193, df = 18.12, *p* = 0.0050) and the ITG (*t* = 3.465, df = 18.08, *p* = 0.0028) of DLB cases compared to controls (Figure 2). A similar pattern was observed for p*α*Syn in the CG (*t* = 4.905, df = 18.01, *p* ≤ 0.0001) and ITG (*t* = 3.858, df = 19.34, *p* = 0.0010) (Figure 2). Measurement of concomitant Alzheimer‐type pathology identified no significant difference in amyloid‐*β* or tau pathology between groups (Figure 3).

**FIGURE 4:**
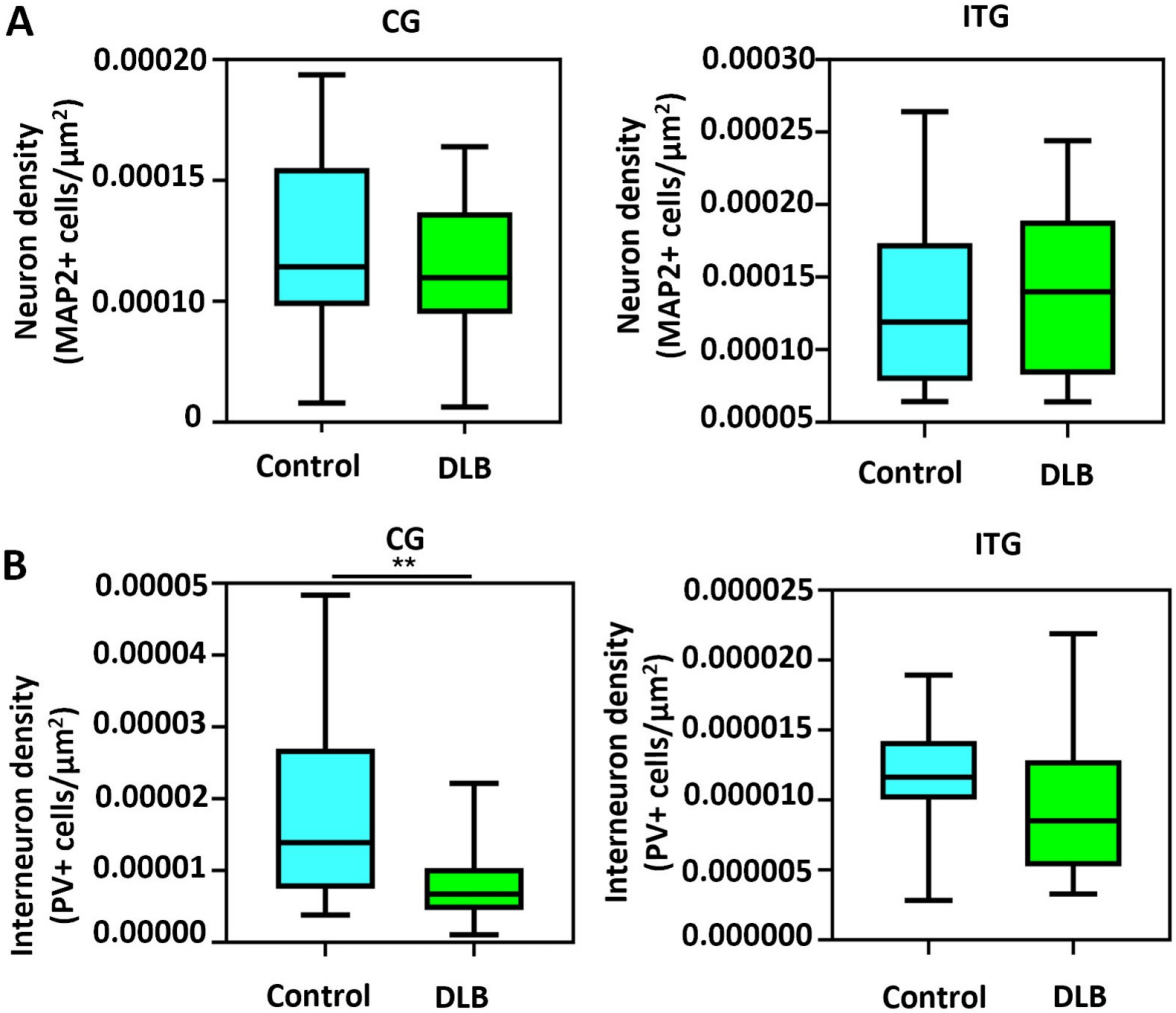
Total neuronal density is not reduced in DLB, but parvalbumin interneurons are of lower abundance in the CG of DLB cases. (A) Neurons counted by MAP 2+ cell bodies in the CG and ITG. (B) Parvalbumin interneurons (PV+) counted in the CG and ITG.

Neuronal density as measured by MAP 2 + neurons did not identify significant differences between DLB and control in either brain region (Figure 4; complete MAP 2 density data are available in Tables S5 and S6). In contrast, parvalbumin interneuron density was significantly lower in DLB CG compared to control (*t* = 2.838, df = 25.86, *p* = 0.0087), but no significant difference was observed between groups in ITG (*p* = 0.1370; Figure 4). Complete parvalbumin neuron density data is available in Tables S8 and S10.

**FIGURE 5:**
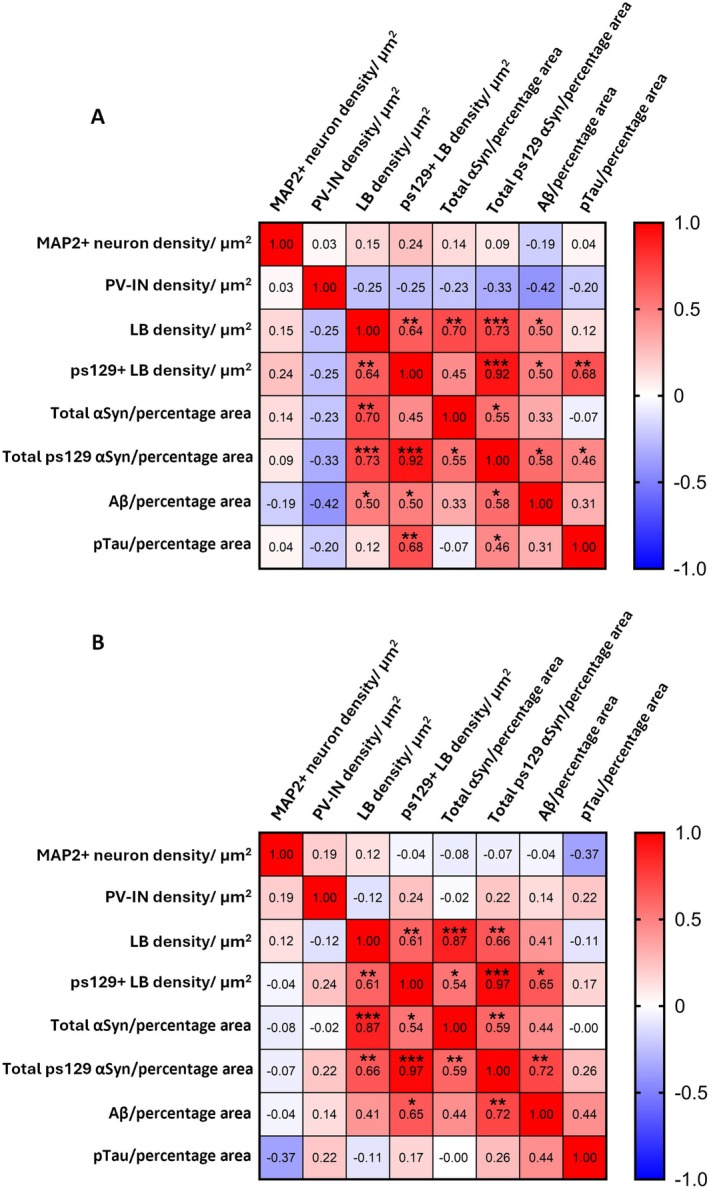
LB density is not associated with neuronal loss in DLB. MAP 2+ neurons, parvalbumin interneurons (PV‐INs), KM51+ and ps129+ LB are calculated as density per μm^2^, and A*β* is presented as percentage area with a size exclusion removing aggregates smaller than 100 μm^2^. *α*Syn, pS129 and pTau are presented as percentage area immunoreactive. (A) Correlations between neuronal density, KM51, ps126 + LB, total *α*Syn, A*β* and pTau pathology in the CG. (B) Correlations between neuronal density, KM51, ps126 + LB, total *α*Syn, A*β* and tau pathology in the ITG.

To probe interrelationships between neuropathological lesions, we performed correlational analyses between the major pathological lesions only in DLB cases to ensure group effects did not identify spurious associations (all data included in Figure 5). In the CG, LB burden as assessed by KM51 was significantly correlated with LB burden as assessed by pS129 (*r* = 0.64, *p* = 0.003) and amyloid‐*β* (*r* = 0.5, *p* = 0.03) but not tau pathology (*p* = 0.627). LB burden assessed by pS129 was correlated with both amyloid‐*β* (*r* = 0.616, *p* = 0.005) and tau pathology (*r* = 0.680, *p* = 0.001) (Figure 5). In the ITG, LB burden as assessed by KM51 was significantly correlated with LB burden as assessed by pS129 (*r* = 0.610, *p* = 0.0060) but not amyloid‐*β* (*r* = 0.413, *p* = 0.0790) or tau (*r* = −0.108, *p* = 0.6605; Figure 5). In the ITG, pS129 + LB burden was significantly correlated with amyloid‐*β* (*r* = 0.62, *p* = 0.008) but not tau pathology (*r* = 0.169, *p* = 0.490). Total *α*Syn and pS129 area immunoreactivity both correlated with LB density, and, as with LB density, pS129 but not KM51 demonstrated relationships with Alzheimer‐type pathology.

### 3.2 Synaptic Marker Abundance in DLB

**FIGURE 6:**
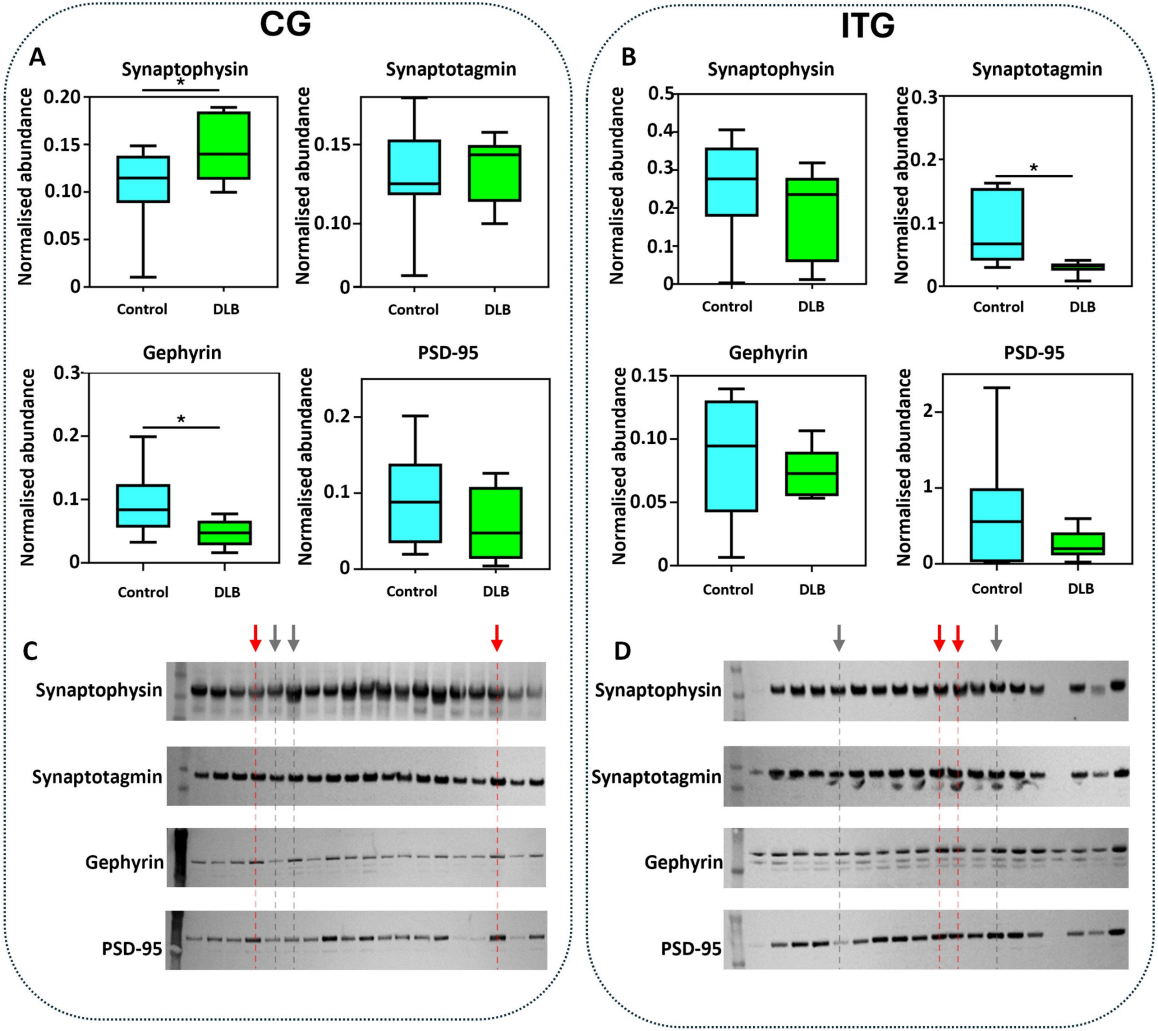
There is no overall decrease in the synaptic proteins synaptophysin, synaptotagmin, gephyrin and PSD‐95 in DLB. (A) Synaptic proteins in the CG, measured by WB (*n* = 10). Proteins were normalised to total proteins as measured by Ponceau. (B) Synaptic proteins in the ITG, measured by WB (*n* = 10). Proteins were normalised to total proteins as measured by Ponceau. (C) Western blots of the pre‐synaptic markers, synaptophysin and synaptotagmin and the postsynaptic markers gephyrin and PSD‐95, in crude brain lysate from CG of DLB cases (*n* = 20). Several samples are highlighted, which contain either very high (red)or very low (grey) KM51+ counts. (D) Pre‐ and post‐synaptic markers in DLB cases in the ITG (*n* = 20). Several samples are highlighted, which contain either very high (red) or very low (grey) LB density. * = *p* value of below 0.05 on a Welch's *t* test.

Immunoblot analysis of synaptic markers in the CG identified that synaptophysin was significantly higher in DLB cases compared to control (*t* = 2.160, df = 17.43, *p* = 0.0450), gephyrin was significantly lower in DLB (*t* = 2.823, df = 12.20, *p* = 0.0152), and there was no significant difference in the abundance of synaptotagmin (*t* = 0.4941, df = 15.92, *p* = 0.6280) or PSD‐95 (*t* = 1.461, df = 16.82, *p* = 0.1625) (Figure 6; full membranes in Figure S4).

In the ITG, synaptotagmin was significantly lower in DLB than controls (*t* = 3.156, df = 9.639, *p* = 0.0107), while there was no difference in abundance of synaptophysin (*t* = 1.074, df = 17.48, *p* = 0.2973), gephyrin (*t* = 0.7470, df = 11.44, *p* = 0.4701) or PSD‐95 (*t* = 1.756, df = 10.25, *p* = 0.1088) (Figure 6; full membranes in Figure S5).

### 3.3 LB Density Is Not Associated With Neuronal or Synaptic Loss

Although no difference was noted in MAP 2+ neuronal density at the group level, we performed an exploratory multiple linear regression analysis to determine whether there was any association between LB burden and neuronal density, including LB density and normalised protein intensity measured by western blot. No association was observed between LBs measured by either KM51 or pS129 in the CG or ITG and neuronal density, with either MAP 2 or parvalbumin, or any synaptic marker, regardless of whether disease duration was included as a co‐variate (Figure 5; full membranes in Figures S6 and S7; regression analyses in Figures [Link], [Link], [Link], [Link], [Link], [Link], [Link], [Link], [Link], [Link], [Link], [Link]). Technical issues with Case 20 in CG and Case 4 in ITG prevented them from being included in this analysis.

In contrast to LB density, total *α*Syn and pS129 area were negatively associated with synaptophysin abundance in the CG, and this relationship was maintained when controlling for disease duration, but no other synaptic proteins exhibited a relationship with *α*Syn or pS129 area (*α*Syn estimate = −0.82, *p* = 0.0069, pS129 estimate = −1.09, *p* = 0.0366; Figures [Link], [Link], [Link], [Link]). Total *α*Syn and pS129 area did not exhibit any associations with neuronal density measured with MAP 2 or parvalbumin in either brain region.

Alzheimer‐type pathology exhibited a similar trend to LB burden, with no significant associations between synaptic proteins and amyloid‐β or hyper‐phosphorylated tau in the CG or ITG (Figure 5).

## 4 Discussion

In the present study, we sought to determine the association between LB pathology and neuronal/synaptic loss in DLB by studying two brain regions, including the CG, a region severely affected by LB in DLB. Despite the putative role ascribed to LB in driving neurodegeneration and synaptic loss in DLB, we did not find any evidence of MAP 2+ neuronal loss and only modest reductions in parvalbumin interneuron density in the CG, gephyrin in the CG, and synaptotagmin in the ITG. These findings are in contrast to the widespread changes in these markers noted in the primary visual cortex in DLB, a region in which LBs do not develop, and we could find no evidence of an association between LB density and neuronal density or synaptic protein abundance in DLB. In contrast to observations with LBs, we found that total *α*Syn and total pS129 immunostaining in the CG were negatively associated with synaptophysin abundance. Taken together, these findings suggest that LB pathology is not clearly or consistently associated with neuronal or synaptic loss in DLB, raising questions about the role played by LB in the neurodegenerative changes thought to drive the phenotype of DLB.

Neuronal loss is thought to be a key pathological driver of neurodegenerative disorders such as DLB. However, the present data suggest that total neuronal density as measured with a pan‐neuronal marker, such as MAP 2, is not significantly lower in DLB cortex compared to controls. Relatively few studies have studied neuronal loss in DLB outside of the brainstem and thalamic nuclei, though we have previously reported no loss of neurons in the primary visual cortex of DLB cases [23]. In contrast, we identified a significantly lower density of parvalbumin interneurons in the CG of DLB cases compared to controls, suggesting sub‐populations of specialised neurons may be vulnerable in DLB. These findings align with a previous report from the DLB hippocampus, where selective loss of parvalbumin interneurons was reported despite no difference in the total number of neurons between groups [28]. This is a notable finding as cortical LBs typically form within pyramidal neurons and not interneurons, thus these observations suggest that neurons which do not manifest LB are selectively vulnerable in the CG [29]. Future studies may wish to evaluate other interneuron subtypes in DLB, such as those labelled by calretinin, calbindin or somatostatin, to determine whether the present observations reflect a generalised vulnerability of interneurons or a specific feature of parvalbumin interneurons. These findings add to the growing neuropathological literature showing no association between LB pathology and neuronal loss, even in regions with significant neuronal loss, such as the substantia nigra [12, 13, 14, 15]. Furthermore, we could not replicate observations from previous studies, which have reported that tau pathology could contribute to neuronal loss in DLB, though it should be noted that our selection criteria precluded cases with the highest levels of Alzheimer‐type pathology to ensure we did not include cases with both DLB and ad [ 30].

Synaptic loss has been widely reported in DLB and related conditions, where it has been associated with cognitive decline [31]. However, the present study found only modestly lower abundance of gephyrin in the CG and synaptotagmin in the ITG, higher abundance of synaptophysin in the CG, and no significant difference in levels of PSD‐95. This is notable given that the CG has a particularly high burden of LB in DLB and, therefore, one would expect particularly marked changes in this region if LB contribute to synaptic loss. Critically, we also identified that there was no association between LB density and any assessed synaptic protein in the CG or ITG, which aligns with a previous study in which the density of LB was not associated with the pre‐synaptic marker, synaptophysin [32]. The modest changes in synaptic proteins in CG and ITG reported here are in contrast to previous observations in the primary visual cortex, a region in which LB do not typically develop, where substantially lower abundance of synaptophysin, PSD‐95 and gephyrin were reported in DLB cases [23]. We have previously reported significantly lower abundance of synaptophysin and PSD‐95 in the pulvinar nucleus of DLB cases compared to controls, despite the pulvinar manifesting only mild *α*Syn pathology [33]. Several other studies in multiple brain regions have reported synaptic protein loss in DLB, though results vary based on the synaptic proteins assessed and the brain regions studied [32, 34, 35]. However, it should be noted that our analysis of synaptic protein abundance only measures the quantity of a given marker within the regions of study, but does not reveal the structure of synapses or their functionality, and a previous study has reported changes to synapse size in DLB [36]. Therefore, it is plausible to suggest that although the abundance of synaptic markers is only modestly changed in DLB, there could be structural and/or functional changes in DLB cases that are not detected by the methods employed. Nevertheless, the lack of clear association between LB density and synaptic loss in both this and previous studies at the very least suggests that LBs are unlikely to be major contributors to synaptic loss in DLB. We did not observe any associations between Alzheimer‐type pathology and synaptic loss, which contrasts with previous findings but could be explained by our decision to only include DLB cases with low or intermediate Alzheimer‐type pathology [7].

In contrast to the poor association between LB and synaptic markers, we observed a negative relationship between the total area immunostained for *α*Syn or pS129 in the CG and the abundance of the pre‐synaptic marker, synaptophysin. This was a notable observation as synaptophysin was significantly increased in the DLB group compared to controls in the CG. Previous studies that have investigated whether *α*Syn pathology associates with synaptic protein loss have generated mixed findings, much of which seems related to variation based on the synaptic proteins evaluated and the regions of the brain studied [34, 37]. Although LBs are often the focus of neuropathological studies, oligomeric species and synaptic aggregates have been proposed to be more toxic, and studies in model systems indicate oligomeric *α*Syn in particular can alter synaptic function [36, 38, 39, 40]. The present observation that only the total area immunostained for *α*Syn, and not LBs alone, correlates with loss of synaptophysin aligns with the view that non‐LB aggregates may be greater contributors to neurodegenerative changes in DLB than LB [4]. Given the advent of new methods to study *α*Syn oligomers in situ*,* which can label objects not typically observed with conventional *α*Syn immunohistochemistry, future studies may wish to build on the present observations and determine whether oligomeric *α*Syn is a better correlate of neuronal and synaptic changes in DLB [38, 41, 42].

Strengths of the present study include our use of clinically and pathologically confirmed DLB cases from a well‐characterised brain bank cohort and our evaluation of neuropathological burden using the entire coronal face of the gyri under evaluation rather than representative sub‐regions, thus minimising bias as much as possible. Nevertheless, we acknowledge some weaknesses in our study, including the histological evaluation of neuropathological burden in one hemisphere and synaptic protein abundance via immunoblotting in the contralateral hemisphere. Although this approach was necessary due to our brain bank dissection protocol, and indeed is an approach used in several other studies evaluating synapses in DLB, an optimal approach would be to evaluate neuropathological and synaptic markers in the same region of the same hemisphere [23, 37]. Nevertheless, it is important to note that presynaptic markers in a given brain region reflect projections onto that region from elsewhere and, therefore, even evaluation of presynaptic markers in the same hemisphere as neuropathology is sub‐optimal. Additionally, although the cohort size is larger than many studies of this nature, and the size of gels for immunoblotting limits the number of cases that can be included, replication of these findings in a larger cohort and across multiple brain regions would be desirable.

In conclusion, the present study has reported no association between LB pathological burden and neuronal or synaptic loss in DLB CG and ITG. In contrast, we observed limited associations between total *α*Syn pathology and synaptophysin loss in the CG. These findings do not support a role for LB formation in key neurodegenerative mechanisms in DLB, questioning the central role ascribed to LB formation in this disorder.

## Author Contributions

Study conception (D.E.); experiments and analysis (J.H., E.K.‐G., S.T., J.O., A.I. and L.W.); writing manuscript (J.H., E.K.‐G. and D.E.); editing manuscript for important intellectual content (S.T., J.O., A.I., L.W. and T.F.O.).

## Funding

This study was funded by the Alzheimer's Research UK (ARUK‐SRF2022A‐006), Deutsche Forschungsgemeinschaft (SFB1286) and the NIHR Newcastle Biomedical Research Centre.

## Ethics Statement

Ethical approval was granted by the NBTR ethical review board (REF: 24/NE/0012, Newcastle and North Tyneside 1 National Health Service [NHS] Research Ethics Committee).

## Conflicts of Interest

The authors declare no conflicts of interest.

## Supporting information


**Table S1:** Antibodies and antibody incubation conditions used in IHC.
**Table S2:** Parameters for LB counts using QuPath.
**Table S3:** LB counts in the CG.
**Table S4:** LB counts in the ITG.
**Table S5:** Neuronal density in the CG, as measured by MAP‐2 + cell bodies.
**Table S6:** Neuronal density in the ITG, as measured by MAP‐2 + cell bodies.
**Table S7:** Parameters for parvalbumin neuron counts using QuPath in the CG.
**Table S8:** Neuronal density in the CG, as measured by parvalbumin+ cell bodies.
**Table S9:** Parameters for parvalbumin neuron counts using QuPath in the ITG.
**Table S10:** Neuronal density in the ITG, as measured by parvalbumin+ cell bodies.
**Table S11:** Protein neuropathology, targeting antibodies and antibody incubation conditions used in Western blots.

**Figure S1:** Sankey diagram demonstrating filtering of the participants to create the current cohort. Case selection was based on a list of all cases in the Newcastle Brain Tissue Resource that were coded as DLB. We then started with the most recent cases and moved back in order of year of death, removing cases that did not fulfil our study criteria until we reached 20 cases. We removed 13 cases, typically for lack of tissue, an atypical clinical presentation, or the presence of significant concomitant pathology, before we obtained sufficient numbers of cases for this study.

**Figure S2:** QuPath thresholding is optimised for individual total αSyn and total pTau slides. A. Manual thresholding for αSyn measured by KM51, where exemplar thresholds of 0.3 and 0.7 are set. B. Manual thresholding for pTau, where exemplar thresholds of 0.2 and 0.3 are set.

**Figure S3:** Disparity between the number of LBs measured using αSyn and pαSyn antibodies. A. LB density in CG in individual cases, comparing KM51 (blue) and ps126 + (orange). B. LB density in ITG in individual cases, comparing KM51 (blue) and ps129 + (orange). *** = p value of below 0.001 on a Welch's t test.

**Figure S4:** Western blots comparing synaptic proteins in the CG in DLB and control. Samples were loaded as follows from left to right: Ladder, DLB20, DLB19, DLB18, DLB17, DLB16, DLB15, DLB14, DLB13, DLB12, DLB11, Control 20, Control19, Control18, Control17, Control16, Control15, Control14, Control13, Control12, Control11. A. Ponceau and Western blots for synaptophysin in the CG. B. Ponceau and Western blots for synaptotagmin in the CG. C. Ponceau and Western blots for Gephyrin in the CG. D. Ponceau and Western blots for PSD‐95 in the CG. Analysed bands are denoted using red arrows.

**Figure S5:** Western blots comparing synaptic proteins in the ITG in DLB and control. Samples were loaded as follows from left to right: Ladder, DLB20, DLB19, DLB18, DLB17, DLB16, DLB15, DLB14, DLB13, DLB12, DLB11, Control 20, Control19, Control18, Control17, Control16, Control15, Control14, Control13, Control12, Control11. A. Ponceau and Western blots for synaptophysin in the ITG. B. Ponceau and Western blots for synaptotagmin in the ITG. C. Ponceau and Western blots for Gephyrin in the ITG. D. Ponceau and Western blots for PSD‐95 in the ITG. Analysed bands are denoted using red arrows.

**Figure S6:** Western blots of all DLB samples used to correlate synaptic proteins with neuropathology in the CG. Samples are loaded as follows from left to right: Ladder, DLB20, DLB19, DLB18, DLB17, DLB16, DLB15, DLB14, DLB13, DLB12, DLB11, DLB10, DLB9, DLB8, DLB7, DLB6, DLB5, DLB4, DLB3, DLB1, Control13, Control1, and Control6. A. Ponceau and Western blots for synaptophysin in the CG. B. Ponceau and Western blots for synaptotagmin in the CG. C. Ponceau and Western blots for Gephyrin in the CG. D. Ponceau and Western blots forPSD‐95 in the CG. Analysed bands are denoted using red arrows.

**Figure S7:** Western blots of all DLB samples used to correlate synaptic proteins with neuropathology in the ITG. Samples are loaded as follows from left to right: Ladder, DLB20, DLB19, DLB18, DLB17, DLB16, DLB15, DLB14, DLB13, DLB12, DLB11, DLB10, DLB9, DLB8, DLB7, DLB6, DLB5, DLB4, DLB3, DLB1, Control13, Control1, and Control6. A. Ponceau and Western blots for synaptophysin in the ITG. B. Ponceau and Western blots for synaptotagmin in the ITG. C. Ponceau and Western blots for Gephyrin in the ITG. D. Ponceau and Western blots for PSD‐95 in the ITG. Analysed bands are denoted using red arrows.

**Figure S8:** Multiple linear regressions of neuronal density against KM51 + and pS129 + LB. MAP 2 + neurons, KM51 + and pS129 + LBs are calculated as density per μm. A. Regression analysis of neuronal density against KM51 + and pS129 + LBs in the CG. B. Regression analysis of neuronal density against KM51 + and pS129 + LBs in the ITG.

**Figure S9:** Multiple linear regressions of neuronal density against KM51 + and pS129 + LB with disease duration as a covariate. MAP 2 + neurons, KM51 + and ps129 + LB are calculated as density per μm. A. Regression analysis of neuronal density against disease duration with KM51 + or pS129 + LB in the CG. B. Regression analysis of neuronal density against disease duration with KM51 + or pS129 + LB in the ITG.

**Figure S10:** Multiple linear regressions of neuronal density against total αSyn and pαSyn by percentage area in the CG and ITG. KM51 and ps129 antibodies were used to calculate total αSyn and pαSyn, inclusive of small inclusions such as Lewy neurites. A. Regression analysis of neuronal density against total αSyn and pαSyn by percentage area in the CG. B. Regression analysis of neuronal density against total αSyn and pαSyn by percentage area in the CG.

**Figure S11:** Multiple linear regressions of neuronal density against total αSyn and pαSyn by percentage area in the CG and ITG, with disease duration added as a covariate. KM51 and ps129 antibodies were used to calculate total αSyn and pαSyn, inclusive of small inclusions such as Lewy neurites. A. Regression analysis of neuronal density against total αSyn and pαSyn by percentage area in the CG. B. Regression analysis of neuronal density against total αSyn and pαSyn by percentage area in the CG.

**Figure S12:** Multiple linear regressions of parvalbumin interneuron density against KM51 + and pS129 + LB. Parvalbumin+ neurons, KM51 + and ps129 + LB are calculated as density per μm. A. Regression analysis of neuronal density against KM51 + and ps129 + LB in the CG. B. Regression analysis of neuronal density against KM51 + and pS129 + LB in the ITG.

**Figure S13:** Multiple linear regressions of parvalbumin interneuron density against KM51 + and pS129 + LB with disease duration as a covariate. Parvalbumin+ neurons, KM51 + and ps129 + LB are calculated as density per μm. A. Regression analysis of neuronal density against disease duration with KM51 + or pS129 + LB in the CG. B. Regression analysis of neuronal density against disease duration with KM51 + or pS129 + LB in the ITG.

**Figure S14:** Multiple linear regressions of parvalbumin interneuron density against total αSyn and pαSyn by percentage area in the CG and ITG. KM51 and ps129 antibodies were used to calculate total αSyn and pαSyn, inclusive of small inclusions such as Lewy neurites. A. Regression analysis of neuronal density against total αSyn and pαSyn by percentage area in the CG. B. Regression analysis of neuronal density against total αSyn and pαSyn by percentage area in the CG.

**Figure S15:** Multiple linear regressions of parvalbumin interneuron density against total αSyn and pαSyn by percentage area in the CG and ITG, with disease duration added as a covariate. KM51 and ps129 antibodies were used to calculate total αSyn and pαSyn, inclusive of small inclusions such as Lewy neurites. A. Regression analysis of neuronal density against total αSyn and pαSyn by percentage area in the CG. B. Regression analysis of neuronal density against total αSyn and pαSyn by percentage area in the CG.

**Figure S16:** Multiple linear regressions of synaptic proteins against KM51 + and pS129 + LBs in CG. KM51 + and pS129 + LB are calculated as density per μm A. KM51 + and pS129 + LB vs. synaptophysin in CG. B. KM51 + and ps129 + LB vs. synaptotagmin in CG. C. KM51 + and pS129 + LB vs. gephyrin in CG. D. KM51 + and ps129 + LB vs. PSD‐95 in CG.

**Figure S17:** Multiple linear regressions of synaptic proteins against KM51 + and pS129 + LBs in ITG. KM51 + and pS129 + LB, calculated as density per μm. A. KM51 + and pS129 + LB vs. synaptophysin in ITG. B. KM51 + and ps129 + LB vs. synaptotagmin in ITG. C. KM51 + and pS129 + LB vs. gephyrin in ITG. D. KM51 + and pS129 + LB vs. PSD‐95 in ITG.

**Figure S18:** Multiple linear regressions of synaptic proteins against KM51 + and pS129 + LBs, as well as disease duration, in CG. KM51 + and ps129 + LB, calculated as density per μm. A. KM51 + and pS129 + LBs vs. synaptophysin in CG. B. KM51 + and pS129 + LBs vs. synaptotagmin in CG. C. KM51 + and pS129 + LBs vs. gephyrin in CG. D. KM51 + and pS129 + LBs vs. PSD‐95 in CG.

**Figure S19:** Multiple linear regressions of synaptic proteins against KM51 + and pS129 + LBs, as well as disease duration, in ITG. KM51 + and pS129 + LBs, calculated as density μm. A. KM51 + and pS129 + LBs vs. synaptophysin in ITG. B. KM51 + and pS129 + LBs vs. synaptotagmin in ITG. C. KM51 + and pS129 + LBs vs. gephyrin in ITG. D. KM51 + and pS129 + LBs vs. PSD‐95 in ITG.

**Figure S20:** Multiple linear regressions of synaptic proteins against total αSyn and pαSyn by percentage area in the CG. KM51 and ps129 antibodies were used to calculate total αSyn and pαSyn, inclusive of small inclusions such as Lewy neurites. A. Total αSyn and pαSyn vs. synaptophysin in CG. B. Total αSyn and pαSyn vs. synaptotagmin in CG. C. Total αSyn and pαSyn vs. gephyrin in CG. D. Total αSyn and pαSyn vs. PSD‐95 in CG.

**Figure S21:** Multiple linear regressions of synaptic proteins against total αSyn and pαSyn by percentage area in the ITG. KM51 and ps129 antibodies were used to calculate total αSyn and pαSyn, inclusive of small inclusions such as Lewy neurites. A. Total αSyn and pαSyn vs. synaptophysin in CG. B. Total αSyn and pαSyn vs. synaptotagmin in CG. C. Total αSyn and pαSyn vs. gephyrin in CG. D. Total αSyn and pαSyn vs. PSD‐95 in CG.

**Figure S22:** Multiple linear regressions of synaptic proteins against total αSyn and pαSyn by percentage area in the CG with disease duration as a covariate. KM51 and ps129 antibodies were used to calculate total αSyn and pαSyn, inclusive of small inclusions such as Lewy neurites. A. Total αSyn and pαSyn vs. synaptophysin in CG. B. Total αSyn and pαSyn vs. synaptotagmin in CG. C. Total αSyn and pαSyn vs. gephyrin in CG. D. Total αSyn and pαSyn vs. PSD‐95 in CG.

**Figure S23:** Multiple linear regressions of synaptic proteins against total αSyn and pαSyn by percentage area in the ITG with disease duration as a covariate. KM51 and pS129 antibodies were used to calculate total αSyn and pαSyn, inclusive of small inclusions such as Lewy neurites. A. Total αSyn and pαSyn vs. synaptophysin in CG. B. Total αSyn and pαSyn vs. synaptotagmin in CG. C. Total αSyn and pαSyn vs. gephyrin in CG. D. Total αSyn and pαSyn vs. PSD‐95 in CG.

**Data S1:** Supporting Information.

**Data S2:** Supporting Information.

## Data Availability

Original data have been included, where possible, in the Supporting Information. Any additional data can be obtained from the corresponding author.
