# Supplementary material for: Lewy Bodies Are Not Associated With Neuronal or Synaptic Loss in Dementia With Lewy Bodies
Source: Neuropathol Appl Neurobiol. 2026 Jun 10;52(3):e70085. doi: 10.1111/nan.70085 (PMC13253057; doi:10.1111/nan.70085)
Supplement: Supplementary file 1 — Table S1: Antibodies and antibody incubation conditions used in IHC. Table S2: Parameters for LB counts using QuPath. Table S3: LB counts in the CG. Table S4: LB counts in the ITG. Table S5: Neuronal density in the CG, as measured by MAP‐2 + cell bodies. Table S6: Neuronal density in the ITG, as measured by MAP‐2 + cell bodies. Table S7: Parameters for parvalbumin neuron counts using QuPath in the CG. Table S8: Neuronal density in the CG, as measured by parvalbumin+ cell bodies. Table S9: Parameters for parvalbumin neuron counts using QuPath in the ITG. Table S10: Neuronal density in the ITG, as measured by parvalbumin+ cell bodies. Table S11: Protein neuropathology, targeting antibodies and antibody incubation conditions used in Western blots. [file NAN-52-e70085-s020.docx]

| **Supporting information Table 1: Antibodies and antibody incubation conditions used in IHC** | | | | |
| --- | --- | --- | --- | --- |
| Target protein | Antibody | Antigen retrieval | Blocking buffer | Dilution |
| αSyn | Leica  ASYN-L | Formulin 10 min at RT  Formic acid 15 min at RT  EDTA 3 min at 95 ͦC | 10% NGS | 1/200 |
| p-αSyn | AbCam  ab51253 | Citrate buffer 10 min at 95 ͦC | 10% NGS | 1/500 |
| Amyloid-beta 4G8 | Covance  SIG-39200 | Formic acid 1 hour | 10% NGS | 1/15000 |
| pTau AT8 | Innogenics | Citrate buffer 10 min at 95 ͦC | 10% NGS | 1/4000 |
| MAP2 | AbCam  ab300645 | Citrate buffer 10 min at 95 ͦC | 10% NGS | 1/10000 |
| Parvalbumin | Sigma  P3088 | Citrate buffer 10 min  at 95 ͦC | 10% NGS | 1/1000 |

| **Supporting information table 2: Parameters for LB counts using QuPath** | |
| --- | --- |
| **Parameter** | **Value** |
| Requested pixel size (µm) | 0.5 |
| Background radius (µm) | 8.0 |
| Opening by reconstruction | True |
| Median radius (µm) | 0.0 |
| Sigma (µm) | 2.0 |
| Min area (µm) | 10.0 |
| Max area (µm) | 1000.0 |
| Threshold | 0.1 |
| Max background | 2.0 |
| Watershed post process | True |
| Exclude DAB | False |
| Cell expansion (µm) | 5.0 |
| Include nuclei | True |
| Smooth boundaries | True |
| Make measurements | True |

| **Supporting information table 3: LB counts in the CG.** | | | | | | |
| --- | --- | --- | --- | --- | --- | --- |
|  | **KM51+ LB** | | | **pS129+ LB** | | |
| **Sample ID** | **Area µm^2^** | **LB count** | **LB/µm2** | **Area µm^2^** | **LB count** | **LB/µm2** |
| **DLB 1** | 6.64E+07 | 109 | 1.64E-06 | 6.30E+07 | 68 | 1.08E-06 |
| **DLB 2** | 4.68E+07 | 200 | 4.27E-06 | 3.19E+07 | 287 | 9.00E-06 |
| **DLB 3** | 4.16E+07 | 234 | 5.63E-06 | 4.51E+07 | 154 | 3.41E-06 |
| **DLB 4** | 3.37E+07 | 227 | 6.74E-06 | 2.36E+07 | 97 | 4.12E-06 |
| **DLB 5** | 2.45E+07 | 11 | 4.50E-07 | 2.52E+07 | 4 | 1.59E-07 |
| **DLB 6** | 5.12E+07 | 36 | 7.04E-07 | 3.91E+07 | 34 | 8.70E-07 |
| **DLB 7** | 5.55E+07 | 12 | 2.16E-07 | 6.01E+07 | 13 | 2.16E-07 |
| **DLB 8** | 5.55E+07 | 88 | 1.59E-06 | 5.70E+07 | 48 | 8.42E-07 |
| **DLB 9** | 6.34E+07 | 187 | 2.95E-06 | 3.68E+07 | 47 | 1.28E-06 |
| **DLB 10** | 2.64E+07 | 92 | 3.49E-06 | 3.29E+07 | 86 | 2.62E-06 |
| **DLB 11** | 5.12E+07 | 63 | 1.23E-06 | 3.24E+07 | 160 | 4.94E-06 |
| **DLB 12** | 5.40E+07 | 174 | 3.22E-06 | 3.75E+07 | 112 | 2.99E-06 |
| **DLB 13** | 2.35E+07 | 30 | 1.28E-06 | 5.54E+07 | 48 | 8.66E-07 |
| **DLB 14** | 6.25E+07 | 92 | 1.47E-06 | 5.01E+07 | 115 | 2.29E-06 |
| **DLB 15** | 9.00E+07 | 288 | 3.20E-06 | 6.90E+07 | 193 | 2.80E-06 |
| **DLB 16** | 5.84E+07 | 260 | 4.45E-06 | 5.94E+07 | 409 | 6.88E-06 |
| **DLB 17** | 2.27E+07 | 204 | 8.98E-06 | 3.38E+07 | 281 | 8.32E-06 |
| **DLB 18** | 2.52E+07 | 68 | 2.70E-06 | 4.54E+07 | 159 | 3.50E-06 |
| **DLB 19** | 4.41E+07 | 284 | 6.44E-06 | 3.51E+07 | 267 | 7.60E-06 |
| **DLB 20** | 3.06E+07 | 89 | 2.91E-06 | 3.02E+07 | 333 | 1.10E-05 |
| **Mean ± SD** | 4.64E+07  ± 1.75E+07 | 137.40  ± 90.08 | 3.18E-06  ± 2.28E-06 | 4.31E+07  ± 1.32E+07 | 145.75  ± 112.50 | 3.74E-06  ± 3.13E-06 |

| **Supporting information table 4: LB counts in the ITG.** | | | | | | |
| --- | --- | --- | --- | --- | --- | --- |
|  | **KM51+ LB** | | | **pS129+ LB** | | |
| **Sample ID** | **Area µm^2^** | **LB count** | **LB/µm2** | **Area µm^2^** | **LB count** | **LB/µm2** |
| **DLB 1** | 4.61E+07 | 25 | 5.42E-07 | 9.37E+07 | 48 | 5.12E-07 |
| **DLB 2** | 3.86E+07 | 98 | 2.54E-06 | 7.07E+07 | 302 | 4.27E-06 |
| **DLB 3** | 3.79E+07 | 52 | 1.37E-06 | 9.28E+07 | 243 | 2.62E-06 |
| **DLB 4** | 4.11E+07 | 118 | 2.87E-06 | 5.99E+07 | 101 | 1.69E-06 |
| **DLB 5** | 1.89E+07 | 1 | 5.30E-08 | 8.88E+07 | 13 | 1.46E-07 |
| **DLB 6** | 5.02E+07 | 53 | 1.06E-06 | 7.18E+07 | 24 | 3.34E-07 |
| **DLB 7** | 3.55E+07 | 3 | 8.46E-08 | 7.34E+07 | 12 | 1.64E-07 |
| **DLB 8** | 6.52E+07 | 23 | 3.53E-07 | 1.18E+08 | 117 | 9.94E-07 |
| **DLB 9** | 6.00E+07 | 10 | 1.67E-07 | 2.89E+07 | 10 | 3.46E-07 |
| **DLB 10** | 7.66E+07 | 321 | 4.19E-06 | 4.31E+07 | 187 | 4.34E-06 |
| **DLB 11** | 3.97E+07 | 187 | 4.71E-06 | 7.86E+07 | 125 | 1.59E-06 |
| **DLB 12** | 2.76E+07 | 65 | 2.35E-06 | 9.54E+07 | 24 | 2.52E-07 |
| **DLB 13** | 4.52E+07 | 2 | 4.42E-08 | 8.56E+07 | 26 | 3.04E-07 |
| **DLB 14** | 6.97E+07 | 31 | 4.45E-07 | 4.14E+07 | 23 | 5.55E-07 |
| **DLB 15** | 5.43E+07 | 32 | 5.89E-07 | 8.14E+07 | 256 | 3.15E-06 |
| **DLB 16** | 7.51E+07 | 109 | 1.45E-06 | 6.15E+07 | 142 | 2.31E-06 |
| **DLB 17** | 7.41E+07 | 113 | 1.53E-06 | 5.08E+07 | 209 | 4.12E-06 |
| **DLB 18** | 1.12E+08 | 38 | 3.40E-07 | 4.18E+07 | 37 | 8.84E-07 |
| **DLB 19** | 2.69E+07 | 103 | 3.83E-06 | 5.52E+07 | 287 | 5.20E-06 |
| **DLB 20** | 2.13E+07 | 17 | 7.99E-07 | 3.22E+07 | 9 | 2.80E-07 |
| **Mean ± SD** | 5.08E+07  ± 2.23E+07 | 70.05  ± 75.29 | 1.47E-06  ± 1.43E-06 | 6.82E+07  ± 2.34E+07 | 109.75  ± 100.53 | 1.70E-06  ± 1.63E-06 |

| **Supporting information table 5: Neuronal density in the CG, as measured by MAP-2+ cell bodies** | | | |
| --- | --- | --- | --- |
| **Sample ID** | **Area µm2** | **Neuronal density (MAP-2+ cell bodies/µm2)** | **Neurons/µm2** |
| **Control 1** | 1.35E+06 | 156 | 1.15E-04 |
| **Control 2** | 1.03E+06 | 177 | 1.71E-04 |
| **Control 3** | 9.72E+05 | 161 | 1.66E-04 |
| **Control 4** | 1.01E+06 | 143 | 1.42E-04 |
| **Control 5** | 1.03E+06 | 101 | 9.78E-05 |
| **Control 6** | 1.56E+06 | 292 | 1.87E-04 |
| **Control 7** | 1.07E+06 | 208 | 1.94E-04 |
| **Control 8** | 1.02E+06 | 101 | 9.89E-05 |
| **Control 9** | 1.54E+06 | 154 | 1.00E-04 |
| **Control 10** | 1.08E+06 | 97 | 8.95E-05 |
| **Control 11** | 1.02E+06 | 153 | 1.50E-04 |
| **Control 12** | 9.86E+05 | 112 | 1.14E-04 |
| **Control 13** | 9.89E+05 | 155 | 1.57E-04 |
| **Control 14** | 1.15E+06 | 169 | 1.46E-04 |
| **Control 15** | 1.14E+06 | 114 | 9.98E-05 |
| **Control 16** | 9.49E+05 | 55 | 5.80E-05 |
| **Control 17** | 9.80E+05 | 63 | 6.43E-05 |
| **Control 18** | 1.47E+06 | 113 | 7.69E-05 |
| **Control 19** | 1.60E+06 | 201 | 1.26E-04 |
| **Control 20** | 1.31E+06 | 143 | 1.09E-04 |
| **DLB 1** | 8.31E+05 | 105 | 1.26E-04 |
| **DLB 2** | 1.04E+06 | 144 | 1.39E-04 |
| **DLB 3** | 1.04E+06 | 124 | 1.20E-04 |
| **DLB 4** | 9.98E+05 | 90 | 9.02E-05 |
| **DLB 5** | 1.18E+06 | 124 | 1.05E-04 |
| **DLB 6** | 1.09E+06 | 118 | 1.08E-04 |
| **DLB 7** | 1.02E+06 | 91 | 8.95E-05 |
| **DLB 8** | 9.52E+05 | 89 | 9.35E-05 |
| **DLB 9** | 9.89E+05 | 162 | 1.64E-04 |
| **DLB 10** | 1.10E+06 | 118 | 1.07E-04 |
| **DLB 11** | 1.00E+06 | 99 | 9.85E-05 |
| **DLB 12** | 1.01E+06 | 152 | 1.50E-04 |
| **DLB 13** | 1.47E+06 | 93 | 6.31E-05 |
| **DLB 14** | 1.14E+06 | 164 | 1.44E-04 |
| **DLB 15** | 1.02E+06 | 114 | 1.12E-04 |
| **DLB 16** | 1.07E+06 | 107 | 1.00E-04 |
| **DLB 17** | 1.00E+06 | 117 | 1.17E-04 |
| **DLB 18** | 1.06E+06 | 60 | 5.63E-05 |
| **DLB 19** | 1.08E+06 | 141 | 1.31E-04 |
| **DLB 20** | 1.09E+06 | 162 | 1.48E-04 |
| **Mean control ± SD** | 1.16E+06 ± 2.15E+05 | 143.40 ± 52.42 | 1.23E-04 ± 3.85E-05 |
| **Mean DLB ± SD** | 1.06E+06 ± 1.18E+05 | 118.70 ± 27.77 | 1.13E-04 ± 2.74E-05 |
| **P value** | 7.28E-02 | 7.74E-02 | 3.60E-01 |

| **Supporting information table 6: Neuronal density in the ITG, as measured by MAP-2+ cell bodies** | | | |
| --- | --- | --- | --- |
| **Sample ID** | **Area µm2** | **Neuronal density (MAP-2+ cell bodies/µm2)** | **Neurons/µm2** |
| **Control 1** | 1.02E+06 | 126 | 1.23E-04 |
| **Control 2** | 9.54E+05 | 173 | 1.81E-04 |
| **Control 3** | 1.05E+06 | 199 | 1.90E-04 |
| **Control 4** | 1.13E+06 | 73 | 6.47E-05 |
| **Control 5** | 1.65E+06 | 344 | 2.09E-04 |
| **Control 6** | 1.10E+06 | 291 | 2.64E-04 |
| **Control 7** | 1.15E+06 | 217 | 1.89E-04 |
| **Control 8** | 1.00E+06 | 150 | 1.49E-04 |
| **Control 9** | 1.02E+06 | 129 | 1.26E-04 |
| **Control 10** | 1.03E+06 | 79 | 7.67E-05 |
| **Control 11** | 6.88E+05 | 63 | 9.16E-05 |
| **Control 12** | 1.24E+06 | 126 | 1.02E-04 |
| **Control 13** | 1.12E+06 | 73 | 6.52E-05 |
| **Control 14** | 1.03E+06 | 155 | 1.51E-04 |
| **Control 15** | 8.14E+05 | 78 | 9.59E-05 |
| **Control 16** | 1.11E+06 | 114 | 6.43E-05 |
| **Control 17** | 1.01E+06 | 146 | 1.44E-04 |
| **Control 18** | 1.04E+06 | 87 | 8.37E-05 |
| **Control 19** | 1.31E+06 | 84 | 6.43E-05 |
| **Control 20** | 1.07E+06 | 123 | 1.15E-04 |
| **DLB 1** | 1.10E+06 | 178 | 1.61E-04 |
| **DLB 2** | 1.00E+06 | 245 | 2.44E-04 |
| **DLB 3** | 1.03E+06 | 66 | 6.40E-05 |
| **DLB 4** | 1.06E+06 | 160 | 1.51E-04 |
| **DLB 5** | 8.42E+05 | 89 | 1.06E-04 |
| **DLB 6** | 1.01E+06 | 192 | 1.90E-04 |
| **DLB 7** | 1.01E+06 | 107 | 1.06E-04 |
| **DLB 8** | 1.03E+06 | 204 | 1.98E-04 |
| **DLB 9** | NA | NA | NA |
| **DLB 10** | 1.29E+06 | 180 | 1.40E-04 |
| **DLB 11** | 9.56E+05 | 155 | 1.62E-04 |
| **DLB 12** | 9.56E+05 | 88 | 9.21E-05 |
| **DLB 13** | 1.00E+06 | 72 | 7.16E-05 |
| **DLB 14** | 1.13E+06 | 78 | 6.91E-05 |
| **DLB 15** | 1.00E+06 | 96 | 9.57E-05 |
| **DLB 16** | 1.23E+06 | 277 | 2.26E-04 |
| **DLB 17** | 1.07E+06 | 166 | 1.55E-04 |
| **DLB 18** | 1.22E+06 | 90 | 7.38E-05 |
| **DLB 19** | 1.00E+06 | 83 | 8.28E-05 |
| **DLB 20** | 1.03E+06 | 194 | 1.89E-04 |
| **Mean Control ± SD** | 1.08E+06 ± 1.83E+05 | 141.50 ± 72.50 | 1.28E-04 ± 5.51E-05 |
| **Mean DLB ± SD** | 1.05E+06 ± 1.02E+05 | 143.16 ± 61.43 | 1.36E-04 ± 5.48E-05 |
| **P value** | 6.09E-01 | 9.41E-01 | 6.55E-01 |

**Supporting information table 7: Parameters for parvalbumin neuron counts using QuPath in the CG**

| **Parameter** | **Value** |
| --- | --- |
| Preferred Pixel Size | 0.35 |
| Resolution | Very High (0.69 µm/px) |
| Channel | DAB |
| Prefilter | Gaussian |
| Smoothing Sigma (µm) | 1 |
| Threshold | 1.15 |
| Above Threshold | Positive |
| Below Threshold | Unclassified |
| Region | Any annotation ROI |
| New Object Type | Annotation |
| Minimum Object Size (µm²) | 45 |
| Minimum Hole Size (µm²) | 0 |
| Split Objects | True |
| Create Objects | True |

| **Supporting information table 8: Neuronal density in the CG, as measured by parvalbumin+ cell bodies** | | | |
| --- | --- | --- | --- |
| **Sample ID** | **Area µm2** | **Parvalbumin+ cell bodies** | **Parvalbumin interneurons/mm2** |
| **Control 1** | 2.46E+06 | 25 | 10.18 |
| **Control 2** | 2.36E+06 | 31 | 13.14 |
| **Control 3** | 2.03E+06 | 17 | 8.37 |
| **Control 4** | 2.42E+06 | 64 | 26.41 |
| **Control 5** | 1.18E+06 | 6 | 5.08 |
| **Control 6** | 9.31E+06 | 253 | 27.18 |
| **Control 7** | 2.35E+06 | 52 | 22.16 |
| **Control 8** | 1.86E+06 | 63 | 33.85 |
| **Control 9** | 1.85E+06 | 27 | 14.61 |
| **Control 10** | 2.15E+06 | 78 | 36.35 |
| **Control 11** | 2.11E+06 | 38 | 18.04 |
| **Control 12** | 1.90E+06 | 92 | 48.35 |
| **Control 13** | 1.65E+06 | 20 | 12.10 |
| **Control 14** | 2.10E+06 | 97 | 46.24 |
| **Control 15** | 2.35E+06 | 9 | 3.83 |
| **Control 16** | 2.39E+06 | 12 | 5.02 |
| **Control 17** | 2.42E+06 | 10 | 4.14 |
| **Control 18** | 3.09E+06 | 23 | 7.44 |
| **Control 19** | 1.59E+06 | 12 | 7.52 |
| **Control 20** | 2.51E+06 | 38 | 15.14 |
| **DLB 1** | 2.09E+06 | 7 | 3.35 |
| **DLB 2** | 2.26E+06 | 5 | 2.22 |
| **DLB 3** | 2.22E+06 | 23 | 10.36 |
| **DLB 4** | 1.88E+06 | 6 | 3.20 |
| **DLB 5** | 2.46E+06 | 48 | 19.54 |
| **DLB 6** | 1.98E+06 | 37 | 18.71 |
| **DLB 7** | 2.73E+06 | 32 | 11.72 |
| **DLB 8** | 2.38E+06 | 24 | 10.10 |
| **DLB 9** | 2.80E+06 | 3 | 1.07 |
| **DLB 10** | 3.53E+06 | 21 | 5.95 |
| **DLB 11** | 1.76E+06 | 8 | 4.54 |
| **DLB 12** | 2.17E+06 | 48 | 22.17 |
| **DLB 13** | 2.48E+06 | 7 | 2.82 |
| **DLB 14** | 2.23E+06 | 15 | 6.71 |
| **DLB 15** | 5.36E+06 | 42 | 7.84 |
| **DLB 16** | 2.34E+06 | 12 | 5.13 |
| **DLB 17** | 3.49E+06 | 30 | 8.60 |
| **DLB 18** | 2.28E+06 | 23 | 10.09 |
| **DLB 19** | 2.44E+06 | 15 | 6.14 |
| **DLB 20** | 1.86E+06 | 12 | 6.47 |
| **Mean Control ± SD** | 2.50E+06 ± 1.65E+06 | 4.84E+01 ± 5.57E+01 | 18.26 ± 13.88 |
| **Mean DLB ± SD** | 2.54E+06 ± 8.12E+05 | 2.09E+01 ± 1.45E+01 | 8.34 ± 55.89 |
| **P value** | 9.39E-01 | 4.45E-02 | 6.82E-03 |

**Supporting information table 9: Parameters for parvalbumin neuron counts using QuPath in the ITG**

| **Parameter** | **Value** |
| --- | --- |
| Preferred Pixel Size | 0.35 |
| Resolution | Very High (0.69 µm/px) |
| Channel | DAB |
| Prefilter | Gaussian |
| Smoothing Sigma (µm) | 1 |
| Threshold | 1.15 |
| Above Threshold | Positive |
| Below Threshold | Unclassified |
| Region | Any annotation ROI |
| New Object Type | Annotation |
| Minimum Object Size (µm²) | 45 |
| Minimum Hole Size (µm²) | 0 |
| Split Objects | True |
| Create Objects | True |

| **Supporting information table 10: Neuronal density in the ITG, as measured by parvalbumin+ cell bodies** | | | |
| --- | --- | --- | --- |
| **Sample ID** | **Area µm2** | **Parvalbumin+ cell bodies** | **Parvalbumin interneurons/mm2** |
| **Control 1** | 1.75E+06 | 25 | 10.18 |
| **Control 2** | 3.22E+06 | 31 | 13.14 |
| **Control 3** | 2.17E+06 | 17 | 8.37 |
| **Control 4** | 2.56E+06 | 64 | 26.41 |
| **Control 5** | 2.93E+06 | 6 | 5.08 |
| **Control 6** | 1.98E+06 | 253 | 27.18 |
| **Control 7** | 2.73E+06 | 52 | 22.16 |
| **Control 8** | 2.66E+06 | 63 | 33.85 |
| **Control 9** | 1.96E+06 | 27 | 14.61 |
| **Control 10** | 2.59E+06 | 78 | 36.35 |
| **Control 11** | 2.37E+06 | 38 | 18.04 |
| **Control 12** | 1.89E+06 | 92 | 48.35 |
| **Control 13** | 2.86E+06 | 20 | 12.10 |
| **Control 14** | 2.12E+06 | 97 | 46.24 |
| **Control 15** | 2.46E+06 | 9 | 3.83 |
| **Control 16** | 1.83E+06 | 12 | 5.02 |
| **Control 17** | 1.83E+06 | 10 | 4.14 |
| **Control 18** | 2.77E+06 | 23 | 7.44 |
| **Control 19** | 1.89E+06 | 12 | 7.52 |
| **Control 20** | 2.24E+06 | 38 | 15.14 |
| **DLB 1** | 2.45E+06 | 7 | 3.35 |
| **DLB 2** | 2.34E+06 | 5 | 2.22 |
| **DLB 3** | 2.06E+06 | 23 | 10.36 |
| **DLB 4** | 2.37E+06 | 6 | 3.20 |
| **DLB 5** | 2.13E+06 | 48 | 19.54 |
| **DLB 6** | 1.92E+06 | 37 | 18.71 |
| **DLB 7** | 2.17E+06 | 32 | 11.72 |
| **DLB 8** | 1.89E+06 | 24 | 10.10 |
| **DLB 9** | 2.82E+06 | 3 | 1.07 |
| **DLB 10** | 2.85E+06 | 21 | 5.95 |
| **DLB 11** | 1.65E+06 | 8 | 4.54 |
| **DLB 12** | 2.44E+06 | 48 | 22.17 |
| **DLB 13** | 3.40E+06 | 7 | 2.82 |
| **DLB 14** | 2.66E+06 | 15 | 6.71 |
| **DLB 15** | 2.10E+06 | 42 | 7.84 |
| **DLB 16** | 2.86E+06 | 12 | 5.13 |
| **DLB 17** | 1.99E+06 | 30 | 8.60 |
| **DLB 18** | 2.57E+06 | 23 | 10.09 |
| **DLB 19** | 1.88E+06 | 15 | 6.14 |
| **DLB 20** | 2.74E+06 | 12 | 6.47 |
| **Mean Control ± SD** | 2.88E+01 ± 1.30E+01 | 2.34E+06 ± 4.35E+05 | 11.97 ± 3.68 |
| **Mean DLB ± SD** | 2.26E+01 ± 1.24E+01 | 2.36E+06 ± 4.36E+05 | 9.85 ± 5.34 |
| **P value** | 1.31E-01 | 8.68E-01 | 1.52E-01 |

| **Supporting information table 11: Protein neuropathology, targeting antibodies and antibody incubation conditions used in Western blots** | | | | | | |
| --- | --- | --- | --- | --- | --- | --- |
| **Target protein** | **Antibody** | **SDS-PAGE running buffer** | **Dilution** | **Blocking buffer** | **Secondary** | **Secondary dilution** |
| Synaptophysin | ProteinTech  17785-1-AP | MES | 1/10000 | 5% BSA | Goat-anti-rabbit (Invitrogen, G21234) | 1/10000 |
| Synaptotagmin | Santa Cruz  sc-136480 | MES | 1/100 | 5% BSA | Goat-anti-mouse (Invitrogen, 31439) | 1/5000 |
| Gephyrin | ProteinTech 12681-1-AP | MOPS | 1/500 | 5% BSA | Goat-anti-rabbit (Invitrogen, G21234) | 1/10000 |
| PSD-95 | ProteinTech  20665-1-AP | MOPS | 1/1000 | 5% BSA | Goat-anti-rabbit (Invitrogen, G21234) | 1/10000 |
