# Supplementary figures and images for: Lewy Bodies Are Not Associated With Neuronal or Synaptic Loss in Dementia With Lewy Bodies
Source: Neuropathol Appl Neurobiol. 2026 Jun 10;52(3):e70085. doi: 10.1111/nan.70085 (PMC13253057; doi:10.1111/nan.70085)

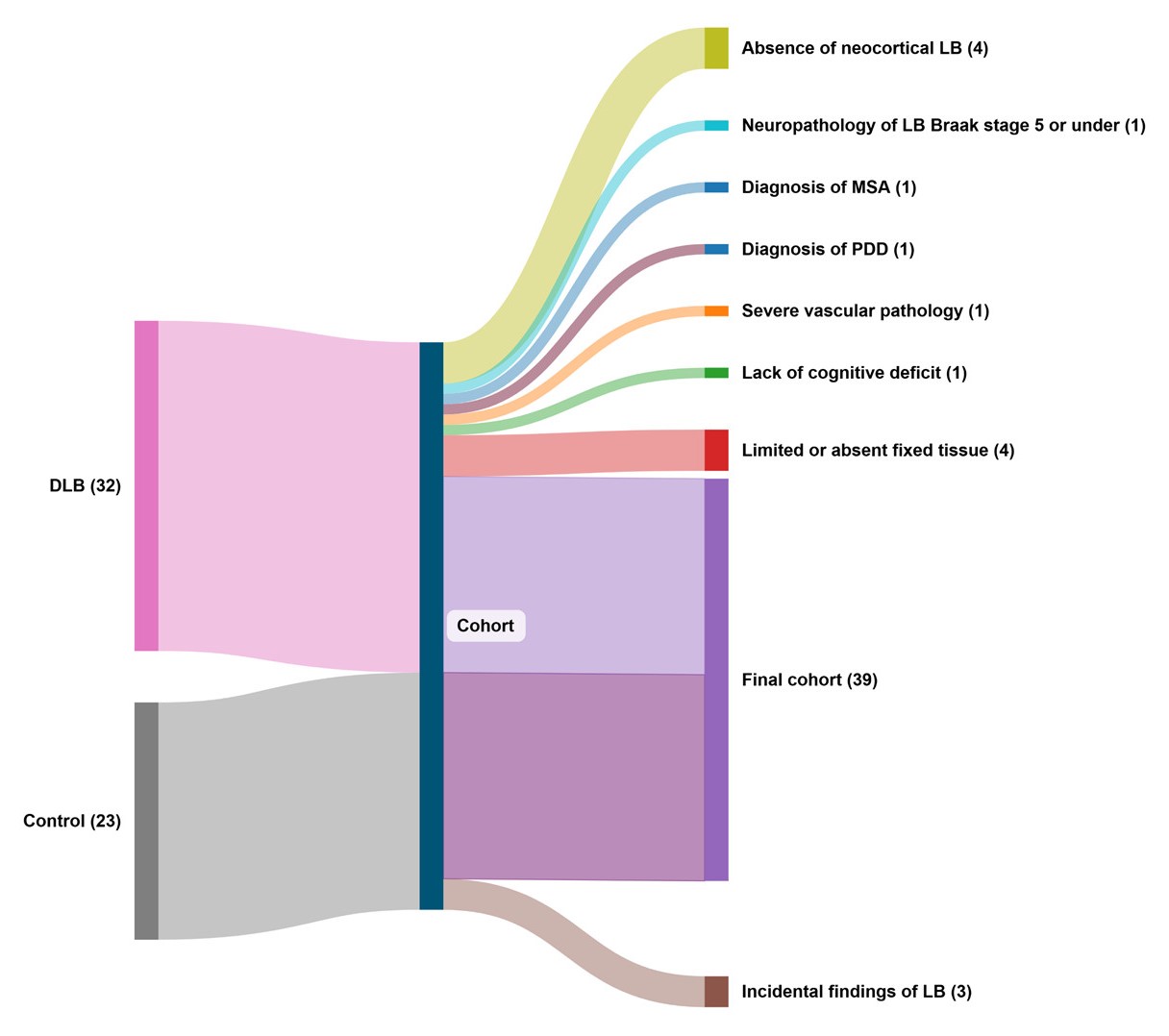

Supplement: Supplementary file 2 — Figure S1: Sankey diagram demonstrating filtering of the participants to create the current cohort. Case selection was based on a list of all cases in the Newcastle Brain Tissue Resource that were coded as DLB. We then started with the most recent cases and moved back in order of year of death, removing cases that did not fulfil our study criteria until we reached 20 cases. We removed 13 cases, typically for lack of tissue, an atypical clinical presentation, or the presence of significant concomitant pathology, before we obtained sufficient numbers of cases for this study. [file NAN-52-e70085-s025.jpg]

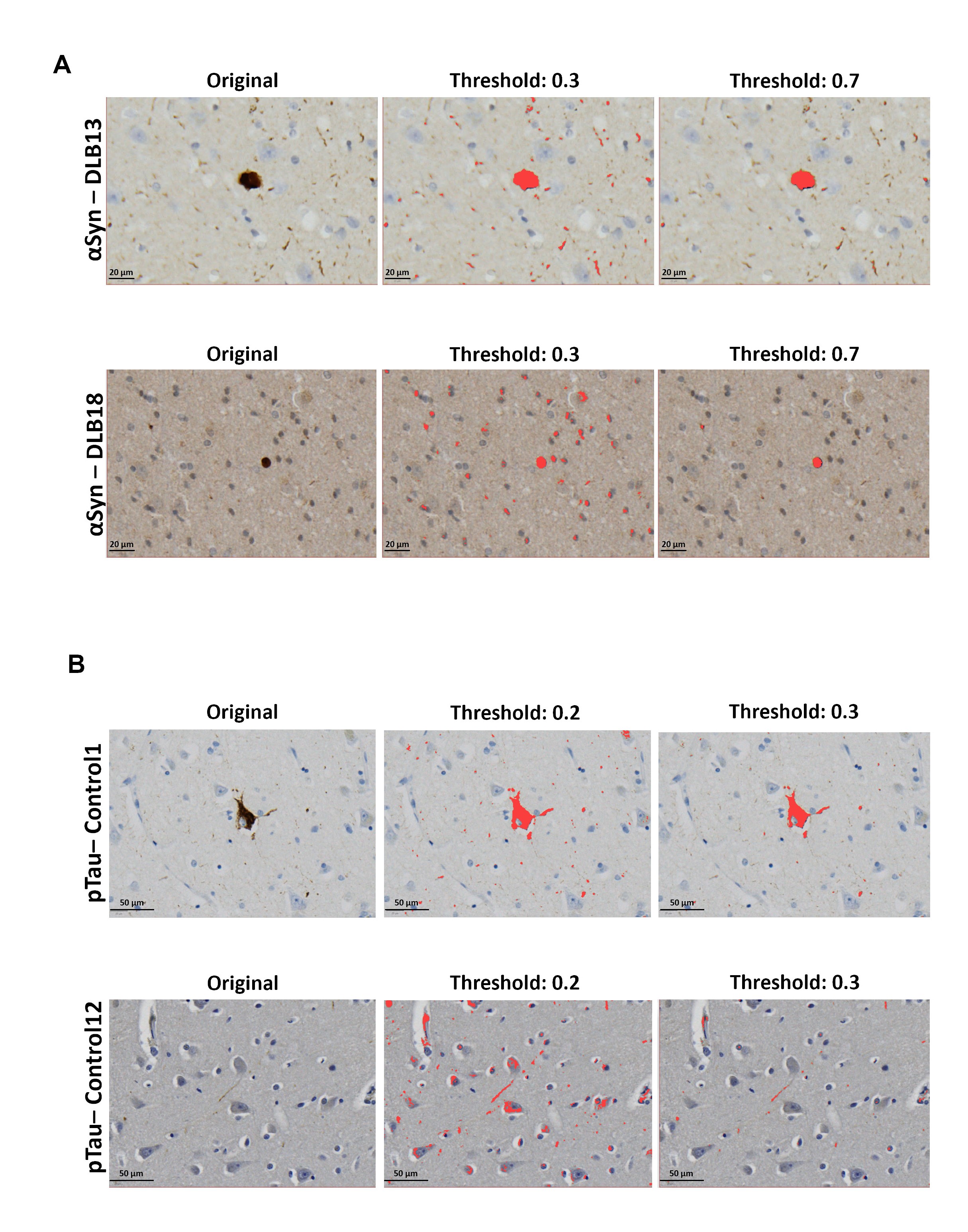

Supplement: Supplementary file 3 — Figure S2: QuPath thresholding is optimised for individual total αSyn and total pTau slides. A. Manual thresholding for αSyn measured by KM51, where exemplar thresholds of 0.3 and 0.7 are set. B. Manual thresholding for pTau, where exemplar thresholds of 0.2 and 0.3 are set. [file NAN-52-e70085-s024.jpg]

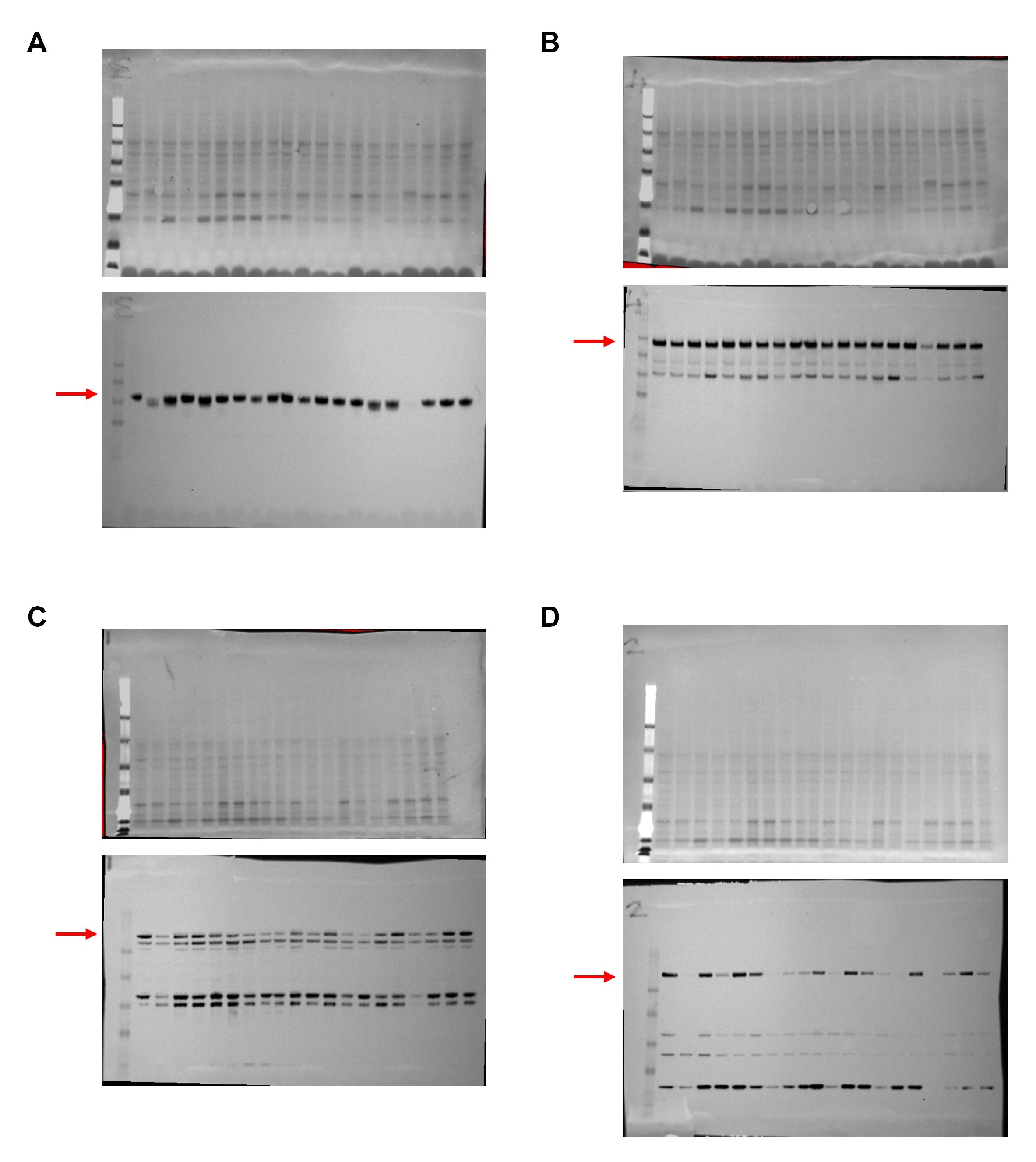

Supplement: Supplementary file 4 — Figure S3: Disparity between the number of LBs measured using αSyn and pαSyn antibodies. A. LB density in CG in individual cases, comparing KM51 (blue) and ps126 + (orange). B. LB density in ITG in individual cases, comparing KM51 (blue) and ps129 + (orange). *** = p value of below 0.001 on a Welch's t test. [file NAN-52-e70085-s006.jpg]

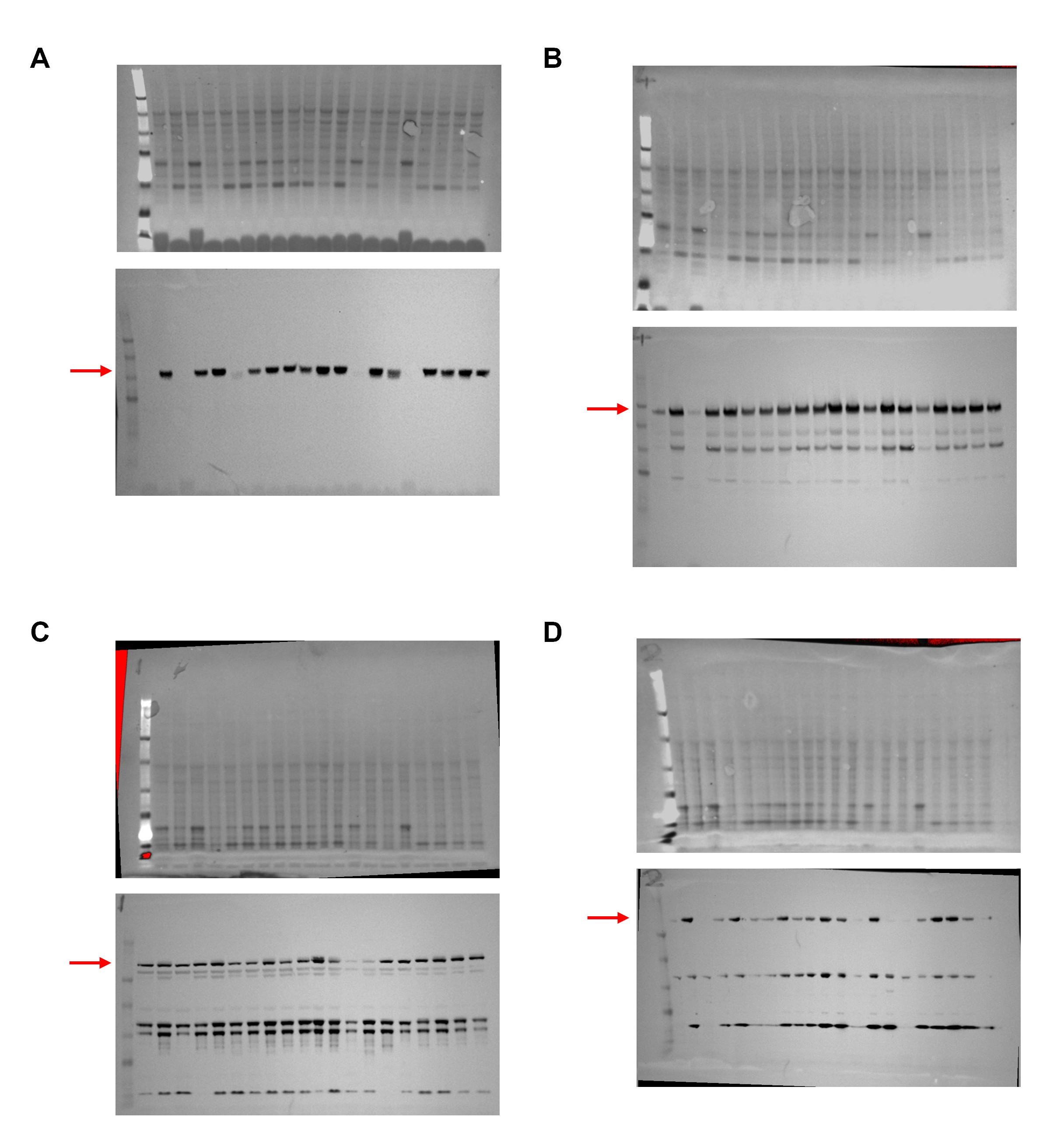

Supplement: Supplementary file 5 — Figure S4: Western blots comparing synaptic proteins in the CG in DLB and control. Samples were loaded as follows from left to right: Ladder, DLB20, DLB19, DLB18, DLB17, DLB16, DLB15, DLB14, DLB13, DLB12, DLB11, Control 20, Control19, Control18, Control17, Control16, Control15, Control14, Control13, Control12, Control11. A. Ponceau and Western blots for synaptophysin in the CG. B. Ponceau and Western blots for synaptotagmin in the CG. C. Ponceau and Western blots for Gephyrin in the CG. D. Ponceau and Western blots for PSD‐95 in the CG. Analysed bands are denoted using red arrows. [file NAN-52-e70085-s004.jpg]

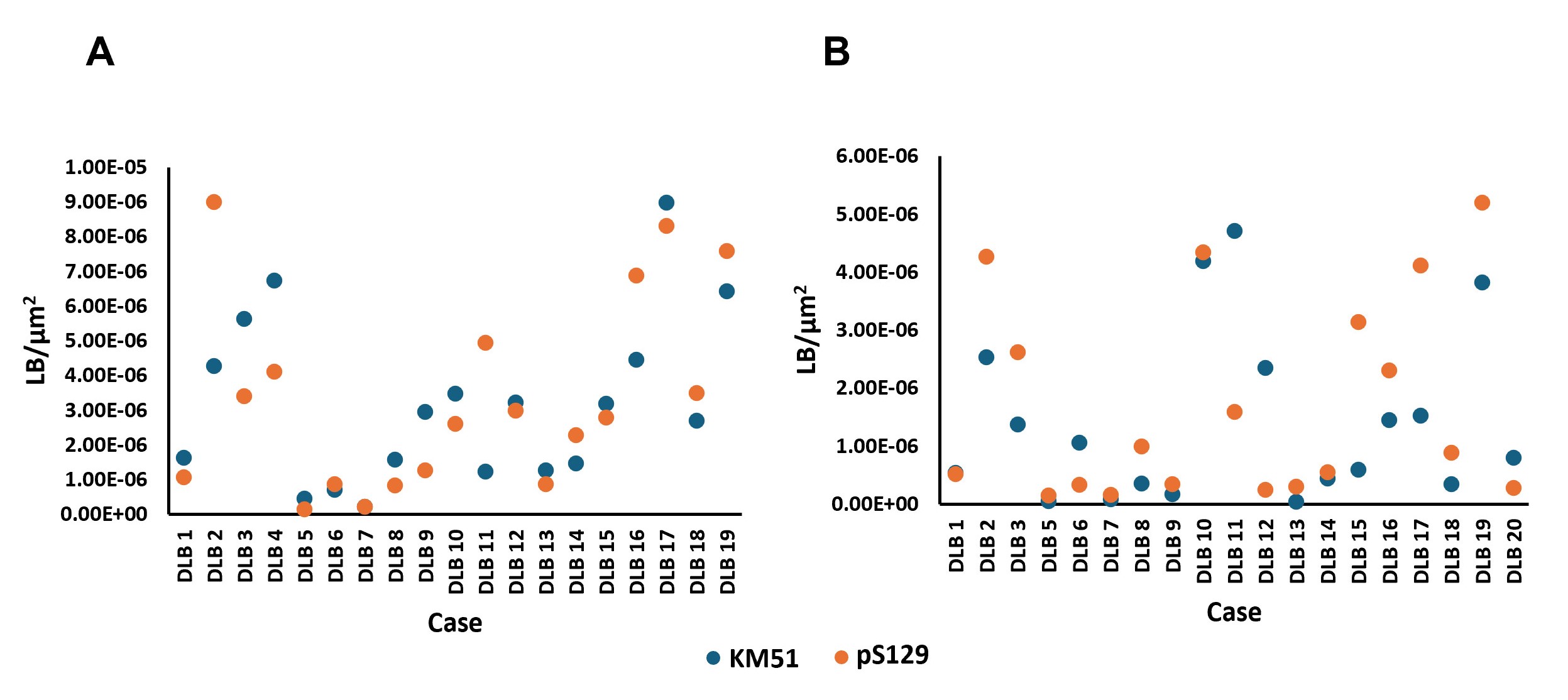

Supplement: Supplementary file 6 — Figure S5: Western blots comparing synaptic proteins in the ITG in DLB and control. Samples were loaded as follows from left to right: Ladder, DLB20, DLB19, DLB18, DLB17, DLB16, DLB15, DLB14, DLB13, DLB12, DLB11, Control 20, Control19, Control18, Control17, Control16, Control15, Control14, Control13, Control12, Control11. A. Ponceau and Western blots for synaptophysin in the ITG. B. Ponceau and Western blots for synaptotagmin in the ITG. C. Ponceau and Western blots for Gephyrin in the ITG. D. Ponceau and Western blots for PSD‐95 in the ITG. Analysed bands are denoted using red arrows. [file NAN-52-e70085-s011.jpg]

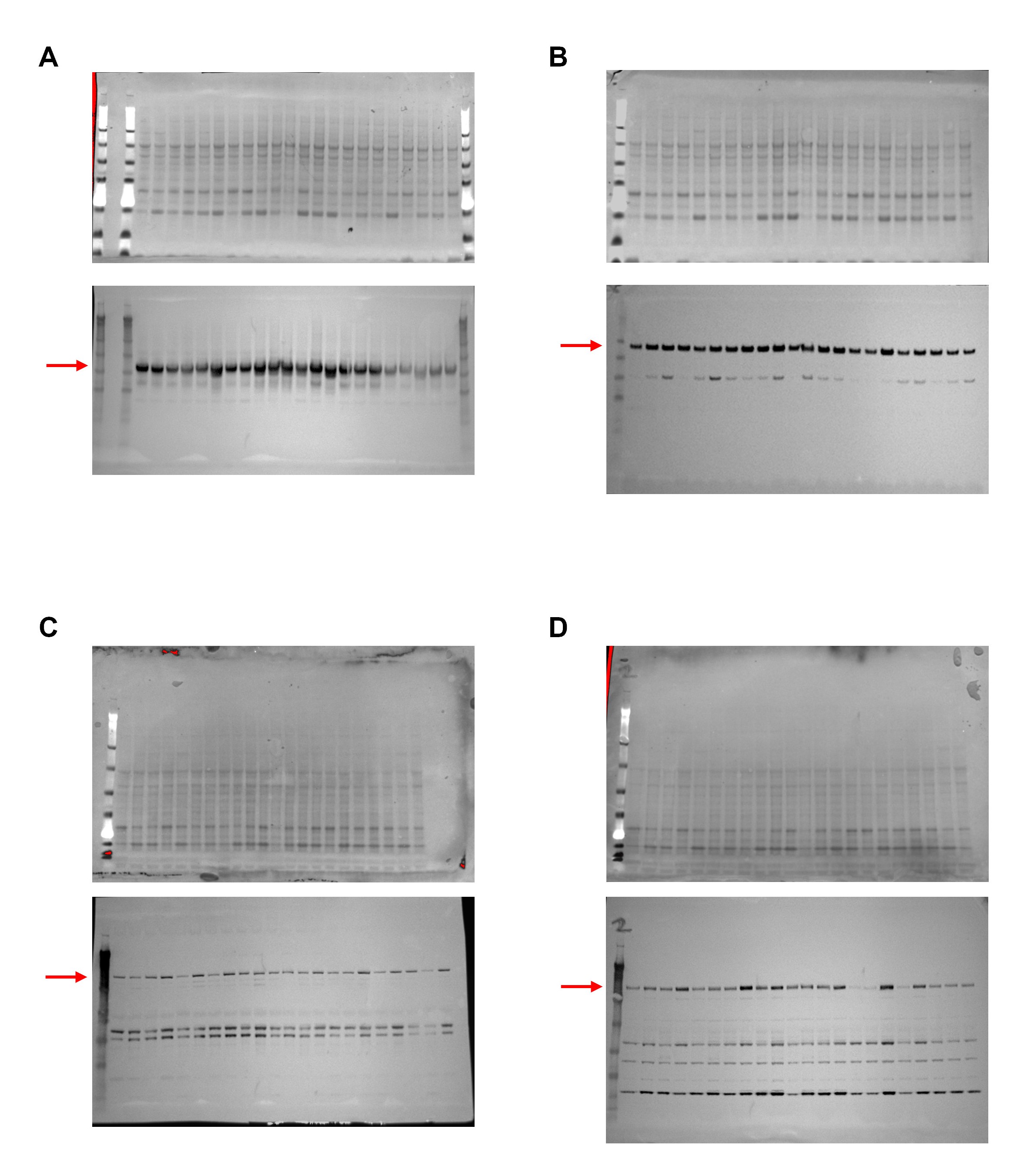

Supplement: Supplementary file 7 — Figure S6: Western blots of all DLB samples used to correlate synaptic proteins with neuropathology in the CG. Samples are loaded as follows from left to right: Ladder, DLB20, DLB19, DLB18, DLB17, DLB16, DLB15, DLB14, DLB13, DLB12, DLB11, DLB10, DLB9, DLB8, DLB7, DLB6, DLB5, DLB4, DLB3, DLB1, Control13, Control1, and Control6. A. Ponceau and Western blots for synaptophysin in the CG. B. Ponceau and Western blots for synaptotagmin in the CG. C. Ponceau and Western blots for Gephyrin in the CG. D. Ponceau and Western blots forPSD‐95 in the CG. Analysed bands are denoted using red arrows. [file NAN-52-e70085-s012.jpg]

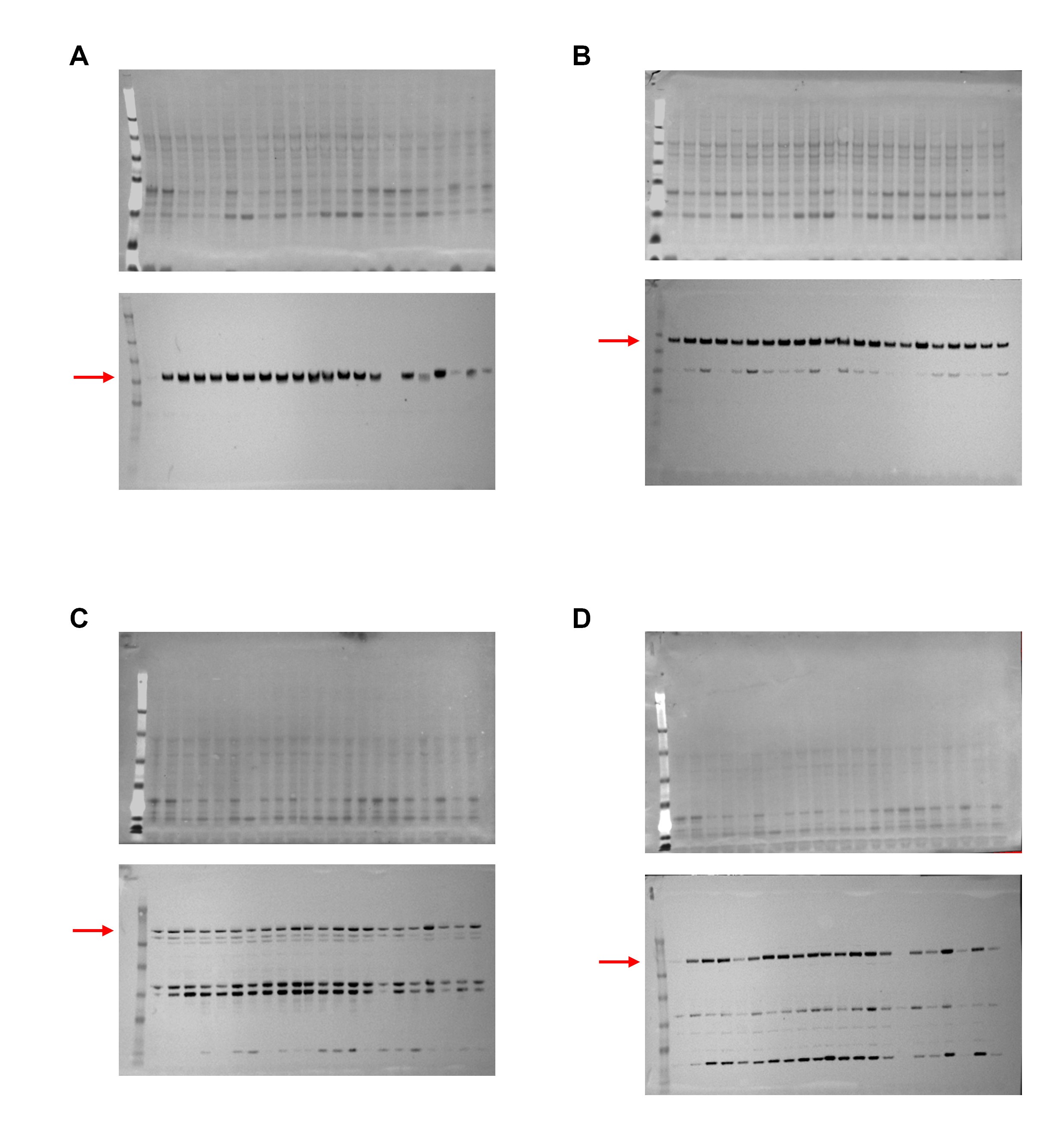

Supplement: Supplementary file 8 — Figure S7: Western blots of all DLB samples used to correlate synaptic proteins with neuropathology in the ITG. Samples are loaded as follows from left to right: Ladder, DLB20, DLB19, DLB18, DLB17, DLB16, DLB15, DLB14, DLB13, DLB12, DLB11, DLB10, DLB9, DLB8, DLB7, DLB6, DLB5, DLB4, DLB3, DLB1, Control13, Control1, and Control6. A. Ponceau and Western blots for synaptophysin in the ITG. B. Ponceau and Western blots for synaptotagmin in the ITG. C. Ponceau and Western blots for Gephyrin in the ITG. D. Ponceau and Western blots for PSD‐95 in the ITG. Analysed bands are denoted using red arrows. [file NAN-52-e70085-s023.jpg]

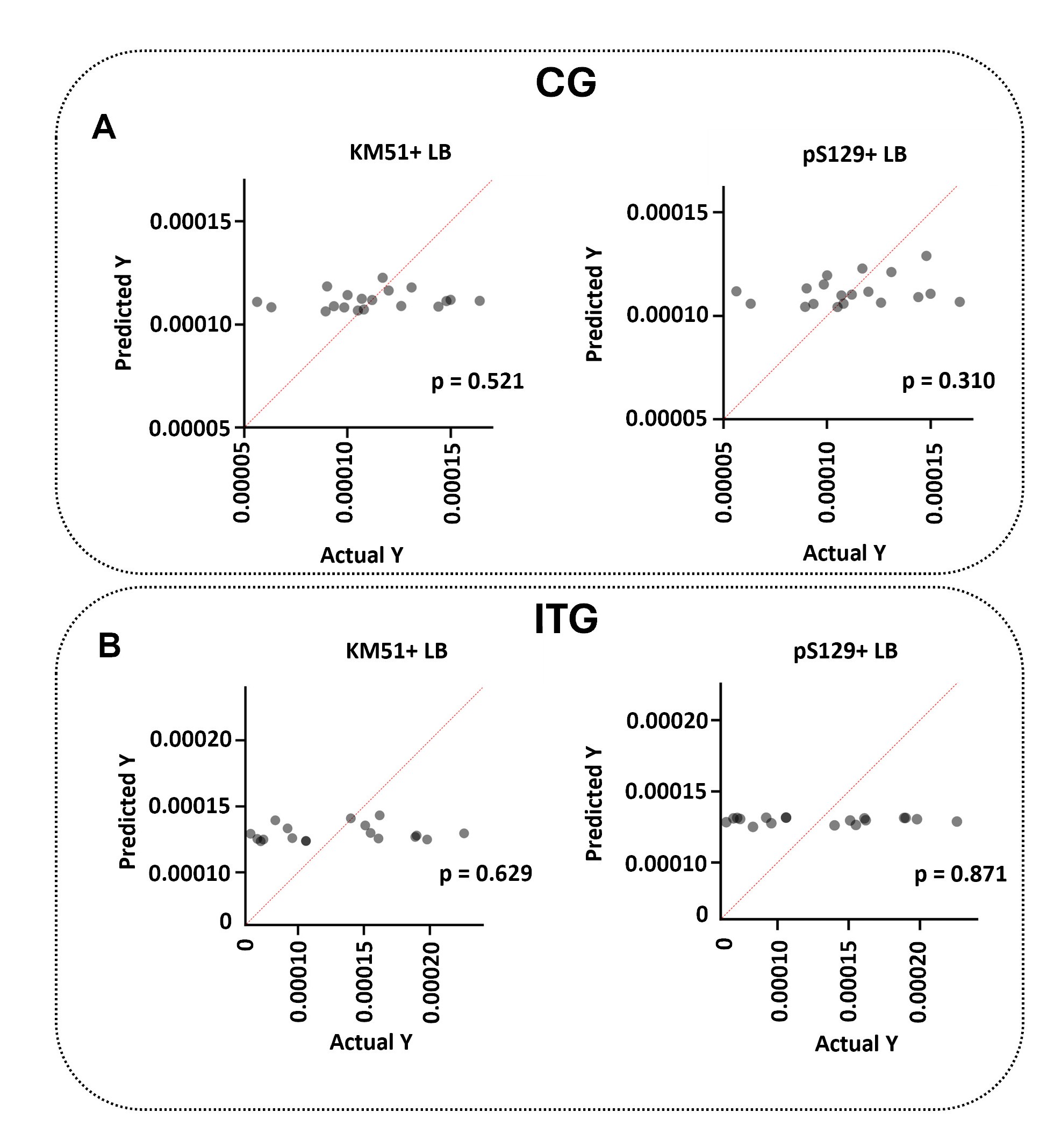

Supplement: Supplementary file 9 — Figure S8: Multiple linear regressions of neuronal density against KM51 + and pS129 + LB. MAP 2 + neurons, KM51 + and pS129 + LBs are calculated as density per μm. A. Regression analysis of neuronal density against KM51 + and pS129 + LBs in the CG. B. Regression analysis of neuronal density against KM51 + and pS129 + LBs in the ITG. [file NAN-52-e70085-s022.jpg]

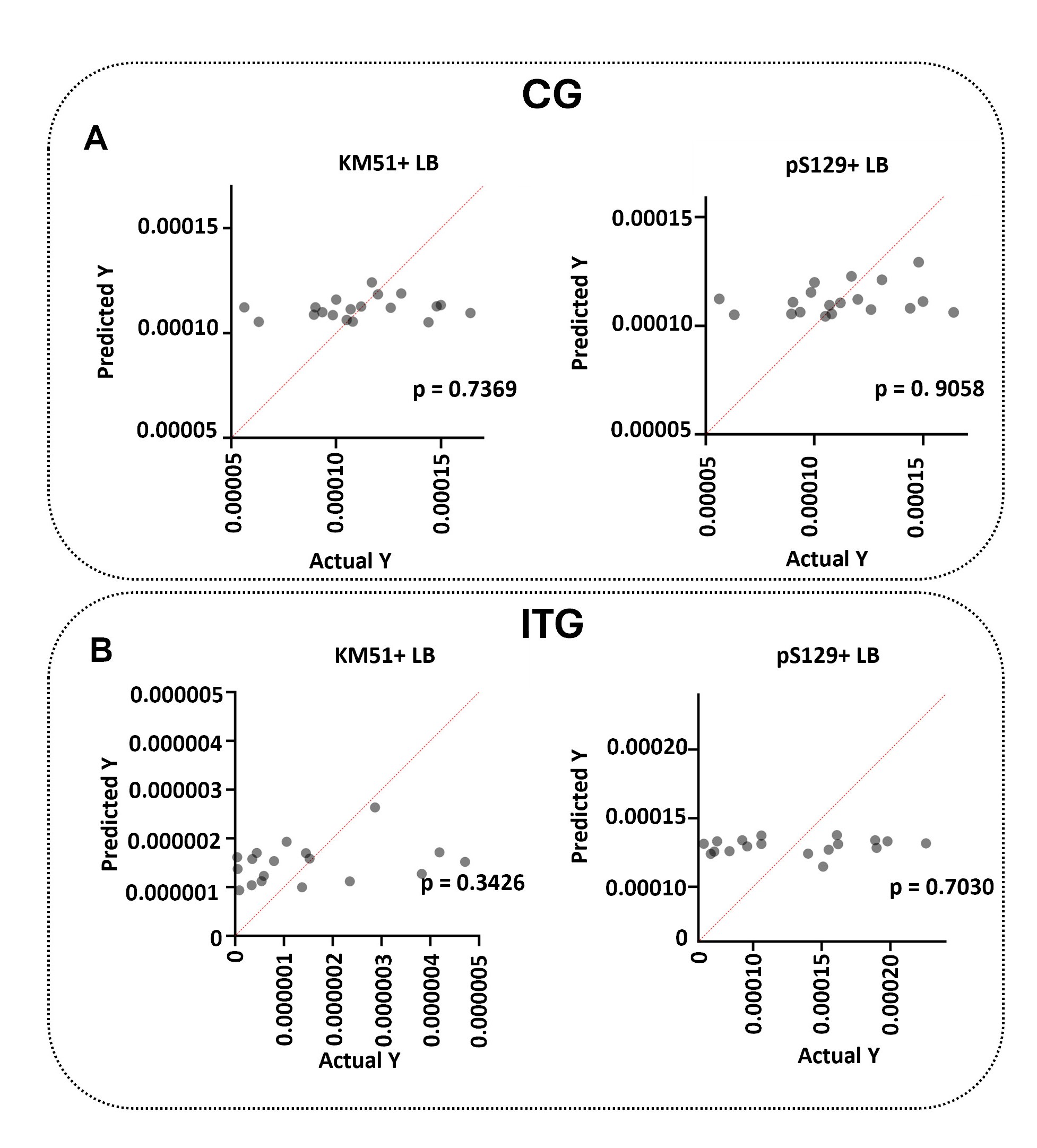

Supplement: Supplementary file 10 — Figure S9: Multiple linear regressions of neuronal density against KM51 + and pS129 + LB with disease duration as a covariate. MAP 2 + neurons, KM51 + and ps129 + LB are calculated as density per μm. A. Regression analysis of neuronal density against disease duration with KM51 + or pS129 + LB in the CG. B. Regression analysis of neuronal density against disease duration with KM51 + or pS129 + LB in the ITG. [file NAN-52-e70085-s001.jpg]

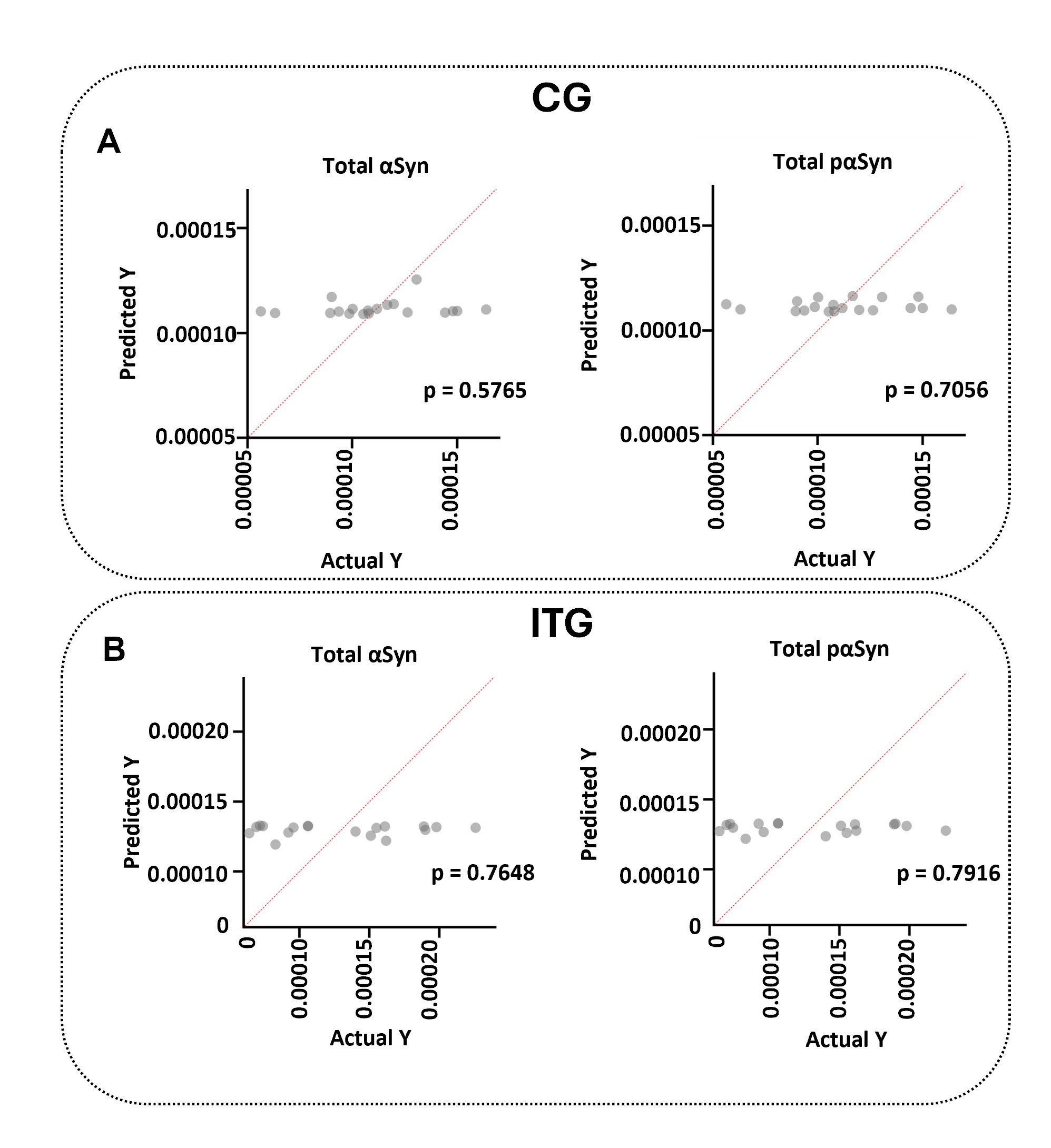

Supplement: Supplementary file 11 — Figure S10: Multiple linear regressions of neuronal density against total αSyn and pαSyn by percentage area in the CG and ITG. KM51 and ps129 antibodies were used to calculate total αSyn and pαSyn, inclusive of small inclusions such as Lewy neurites. A. Regression analysis of neuronal density against total αSyn and pαSyn by percentage area in the CG. B. Regression analysis of neuronal density against total αSyn and pαSyn by percentage area in the CG. [file NAN-52-e70085-s005.jpg]

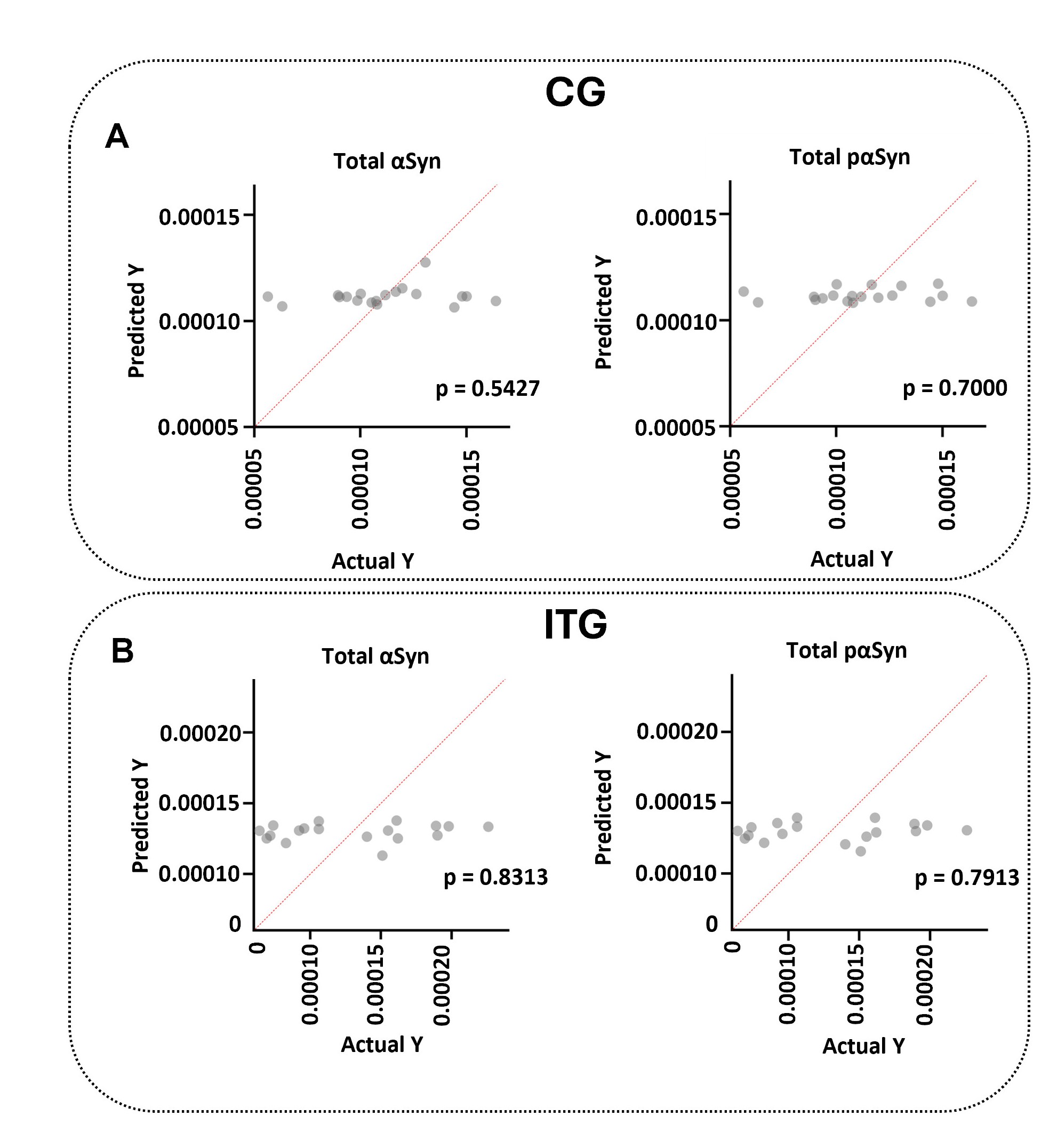

Supplement: Supplementary file 12 — Figure S11: Multiple linear regressions of neuronal density against total αSyn and pαSyn by percentage area in the CG and ITG, with disease duration added as a covariate. KM51 and ps129 antibodies were used to calculate total αSyn and pαSyn, inclusive of small inclusions such as Lewy neurites. A. Regression analysis of neuronal density against total αSyn and pαSyn by percentage area in the CG. B. Regression analysis of neuronal density against total αSyn and pαSyn by percentage area in the CG. [file NAN-52-e70085-s003.jpg]

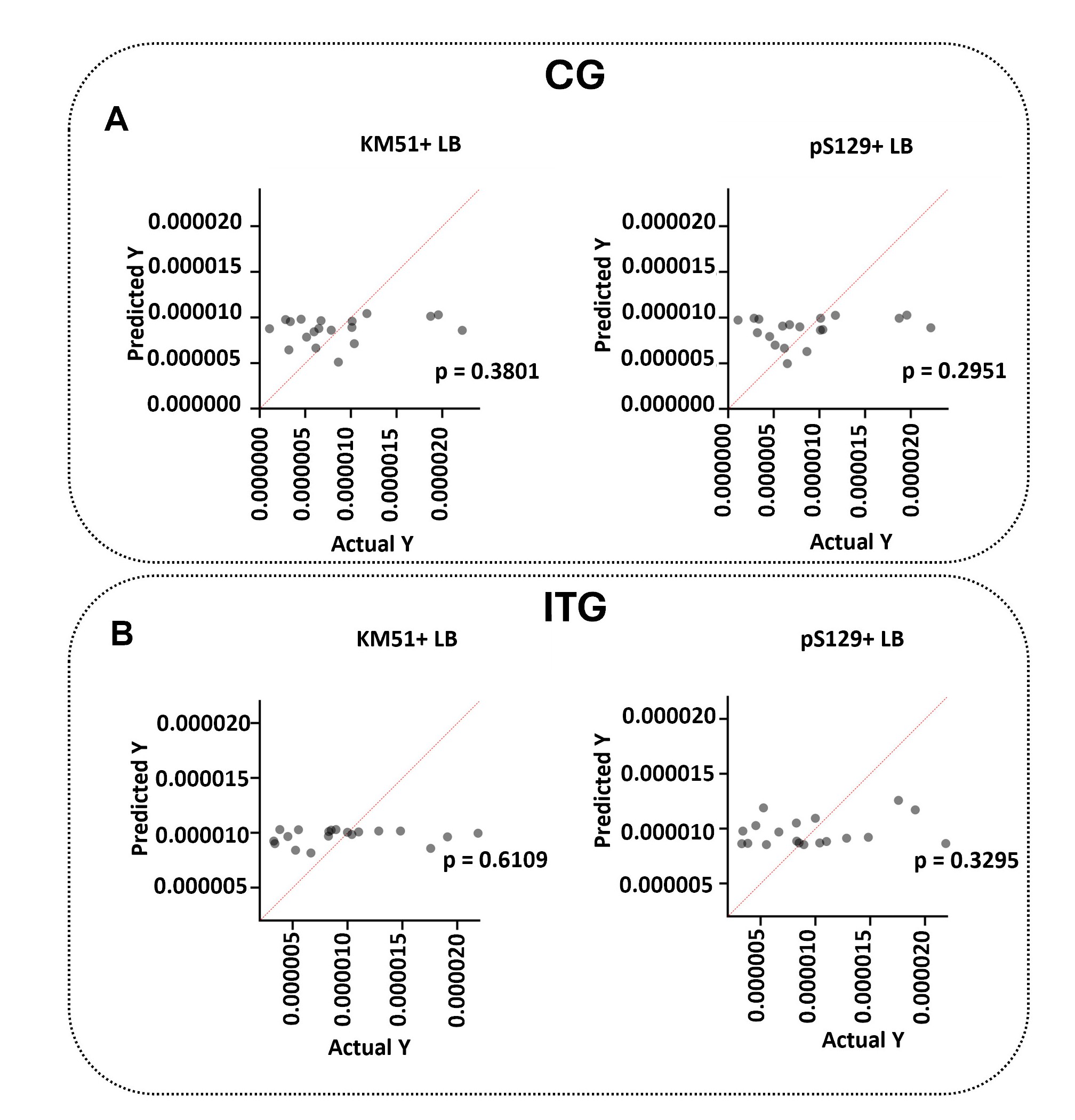

Supplement: Supplementary file 13 — Figure S12: Multiple linear regressions of parvalbumin interneuron density against KM51 + and pS129 + LB. Parvalbumin+ neurons, KM51 + and ps129 + LB are calculated as density per μm. A. Regression analysis of neuronal density against KM51 + and ps129 + LB in the CG. B. Regression analysis of neuronal density against KM51 + and pS129 + LB in the ITG. [file NAN-52-e70085-s019.jpg]

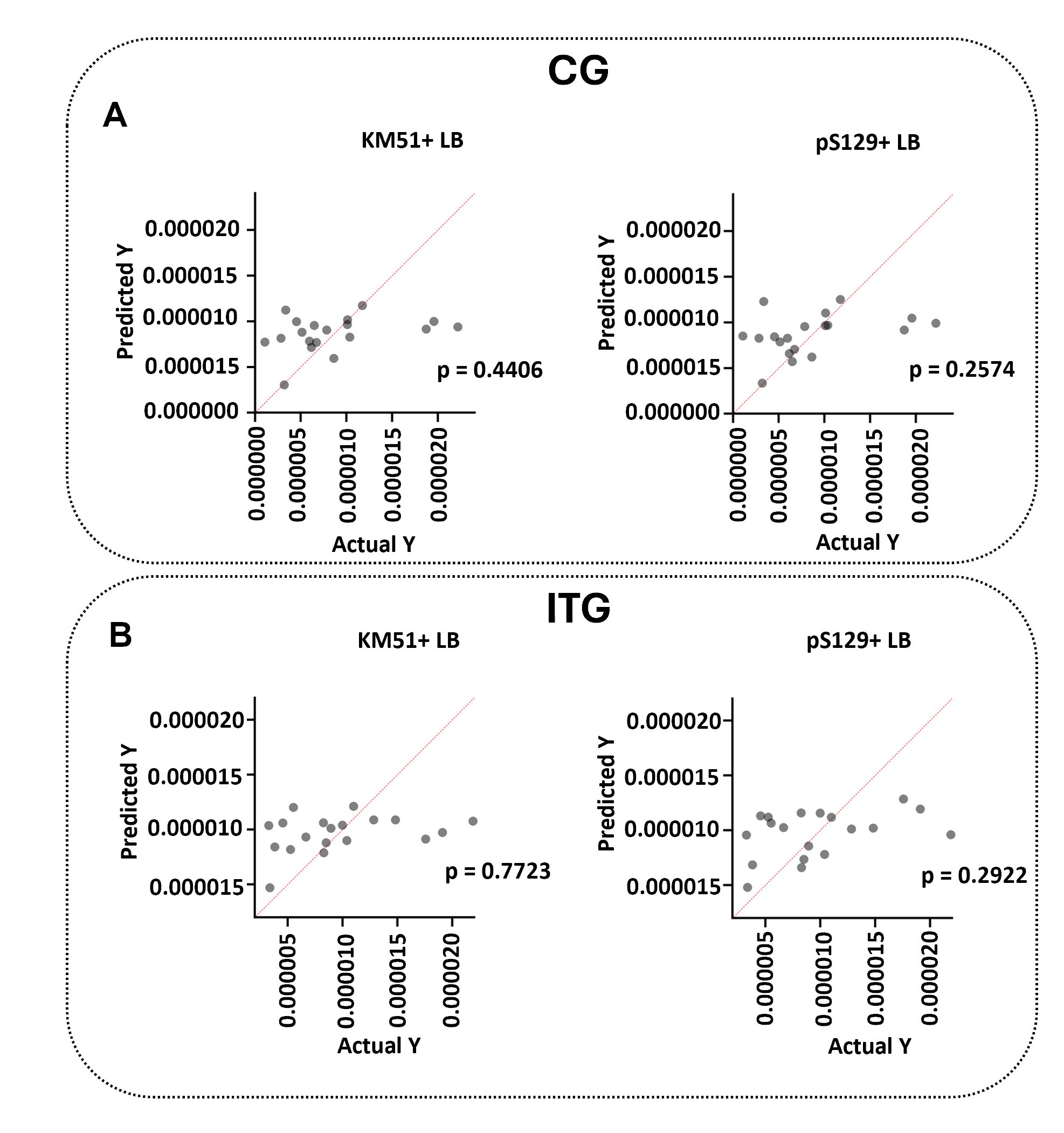

Supplement: Supplementary file 14 — Figure S13: Multiple linear regressions of parvalbumin interneuron density against KM51 + and pS129 + LB with disease duration as a covariate. Parvalbumin+ neurons, KM51 + and ps129 + LB are calculated as density per μm. A. Regression analysis of neuronal density against disease duration with KM51 + or pS129 + LB in the CG. B. Regression analysis of neuronal density against disease duration with KM51 + or pS129 + LB in the ITG. [file NAN-52-e70085-s008.jpg]

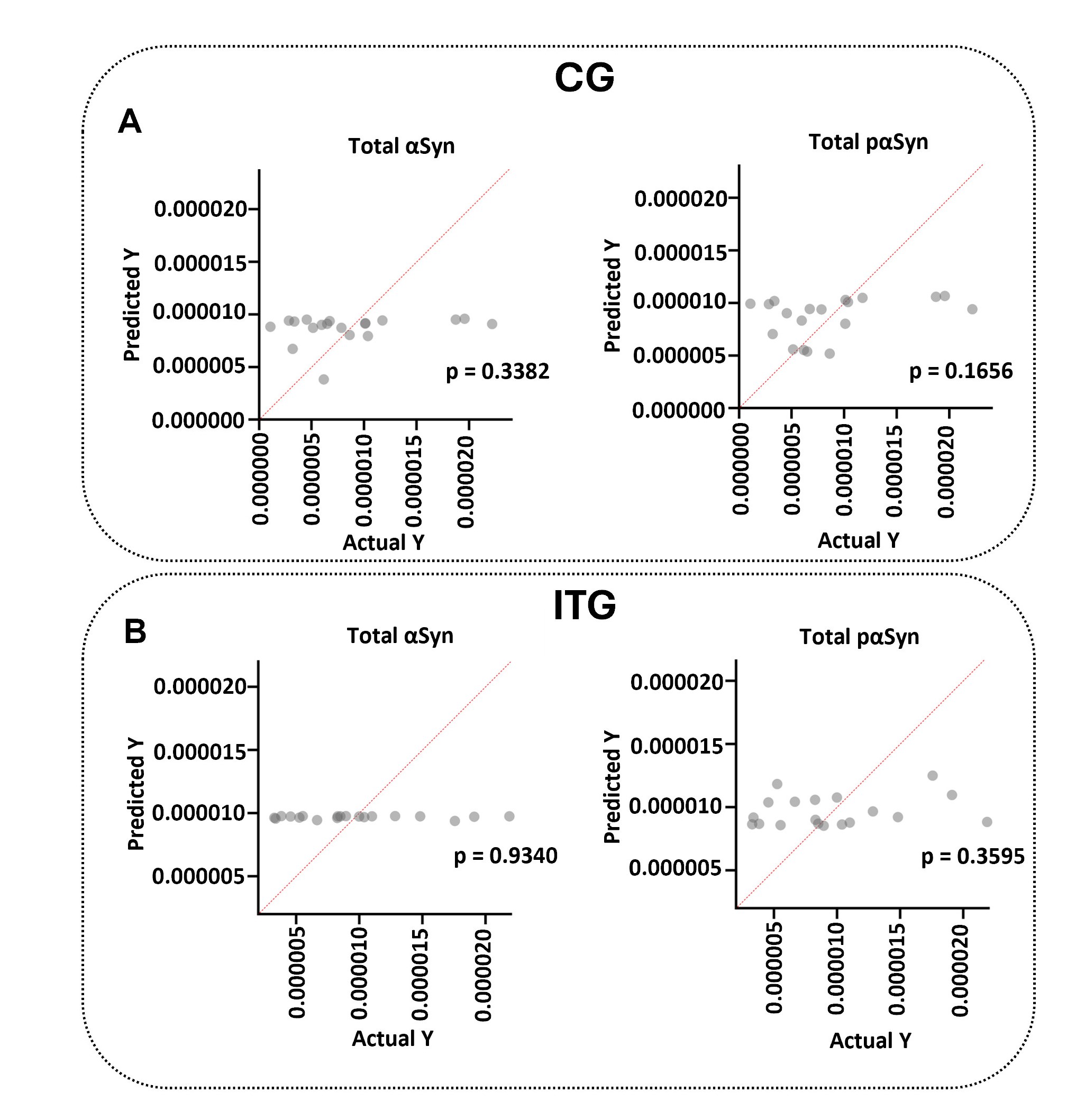

Supplement: Supplementary file 15 — Figure S14: Multiple linear regressions of parvalbumin interneuron density against total αSyn and pαSyn by percentage area in the CG and ITG. KM51 and ps129 antibodies were used to calculate total αSyn and pαSyn, inclusive of small inclusions such as Lewy neurites. A. Regression analysis of neuronal density against total αSyn and pαSyn by percentage area in the CG. B. Regression analysis of neuronal density against total αSyn and pαSyn by percentage area in the CG. [file NAN-52-e70085-s009.jpg]

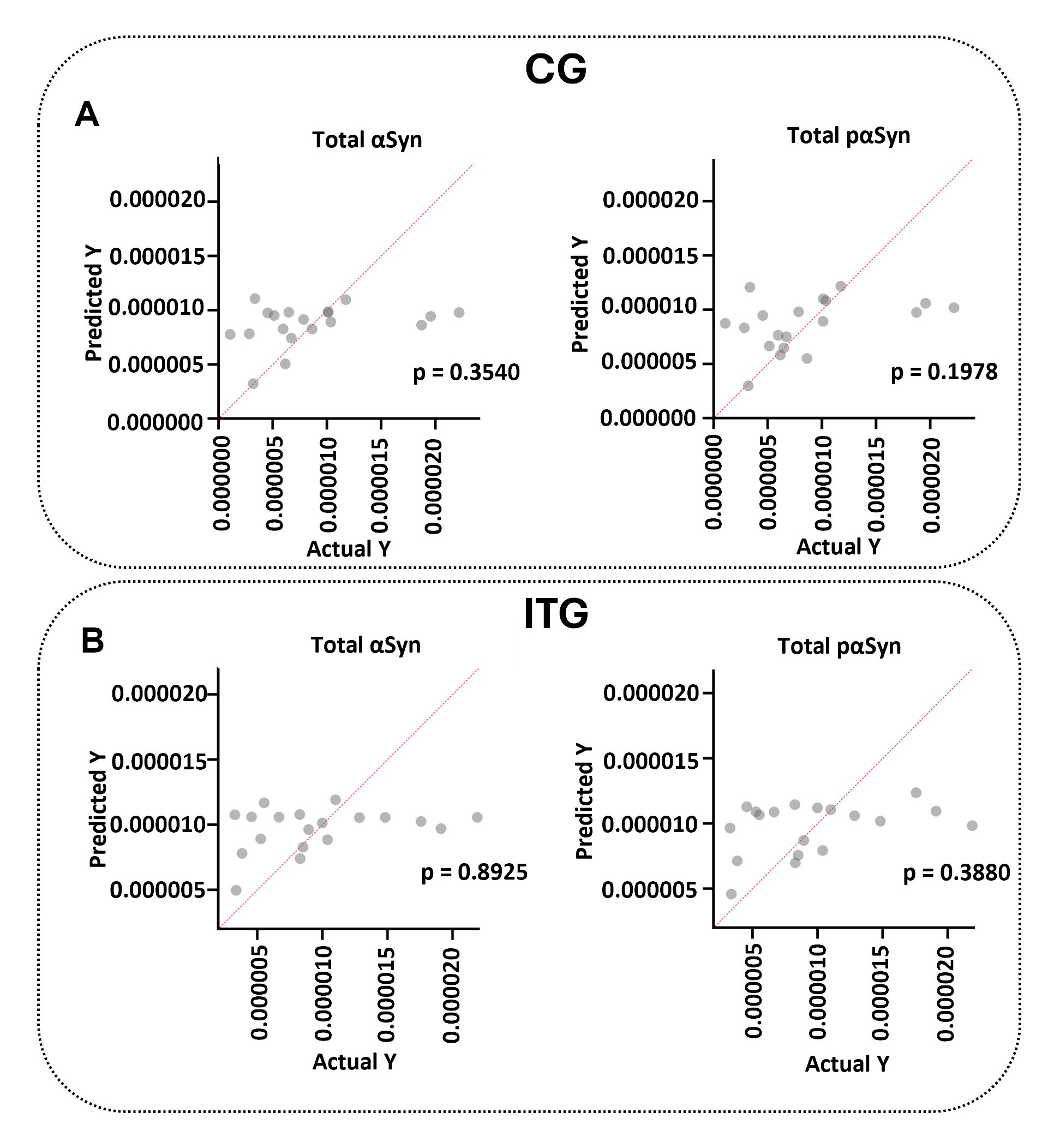

Supplement: Supplementary file 16 — Figure S15: Multiple linear regressions of parvalbumin interneuron density against total αSyn and pαSyn by percentage area in the CG and ITG, with disease duration added as a covariate. KM51 and ps129 antibodies were used to calculate total αSyn and pαSyn, inclusive of small inclusions such as Lewy neurites. A. Regression analysis of neuronal density against total αSyn and pαSyn by percentage area in the CG. B. Regression analysis of neuronal density against total αSyn and pαSyn by percentage area in the CG. [file NAN-52-e70085-s007.jpg]

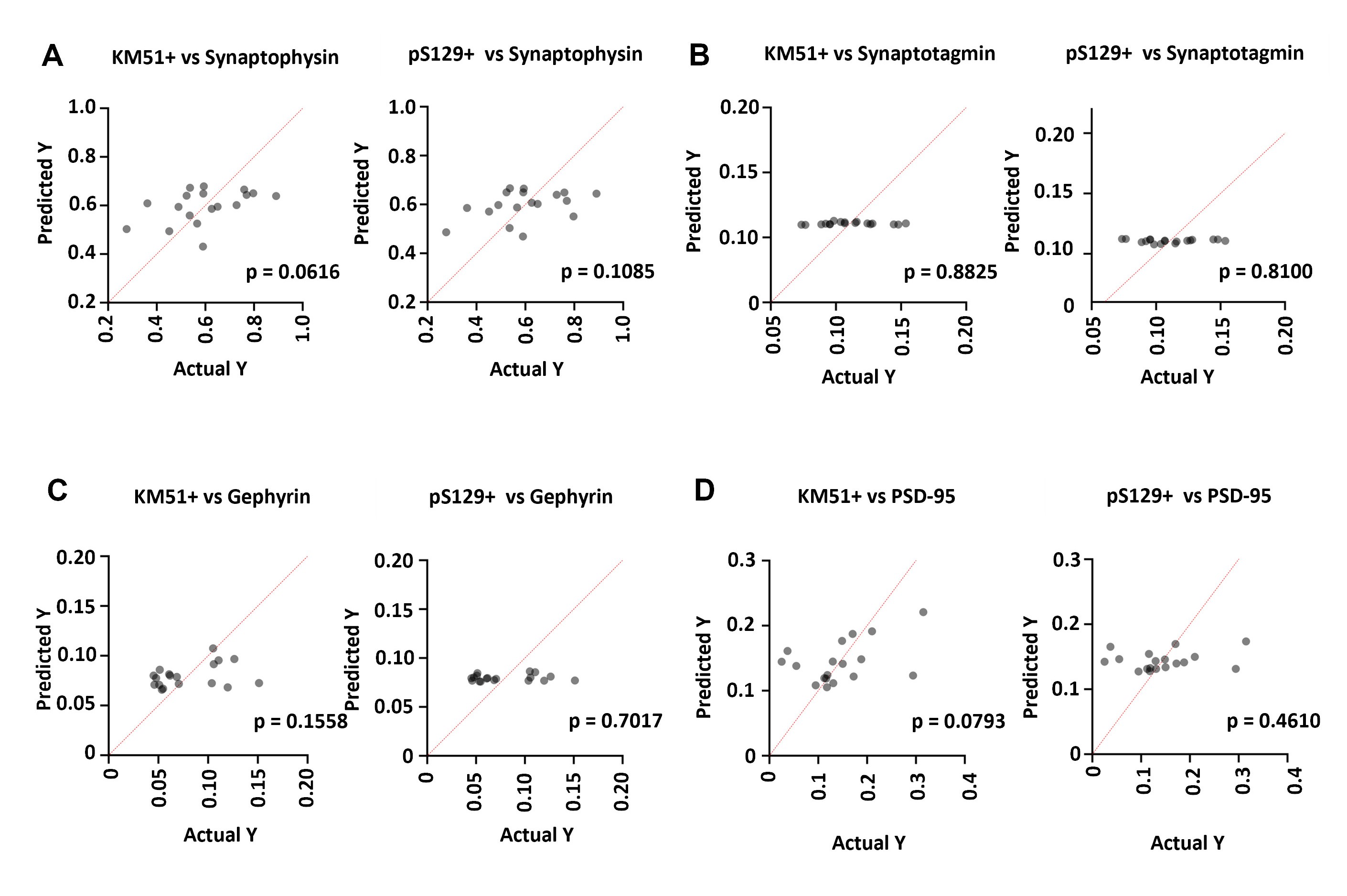

Supplement: Supplementary file 17 — Figure S16: Multiple linear regressions of synaptic proteins against KM51 + and pS129 + LBs in CG. KM51 + and pS129 + LB are calculated as density per μm A. KM51 + and pS129 + LB vs. synaptophysin in CG. B. KM51 + and ps129 + LB vs. synaptotagmin in CG. C. KM51 + and pS129 + LB vs. gephyrin in CG. D. KM51 + and ps129 + LB vs. PSD‐95 in CG. [file NAN-52-e70085-s014.jpg]

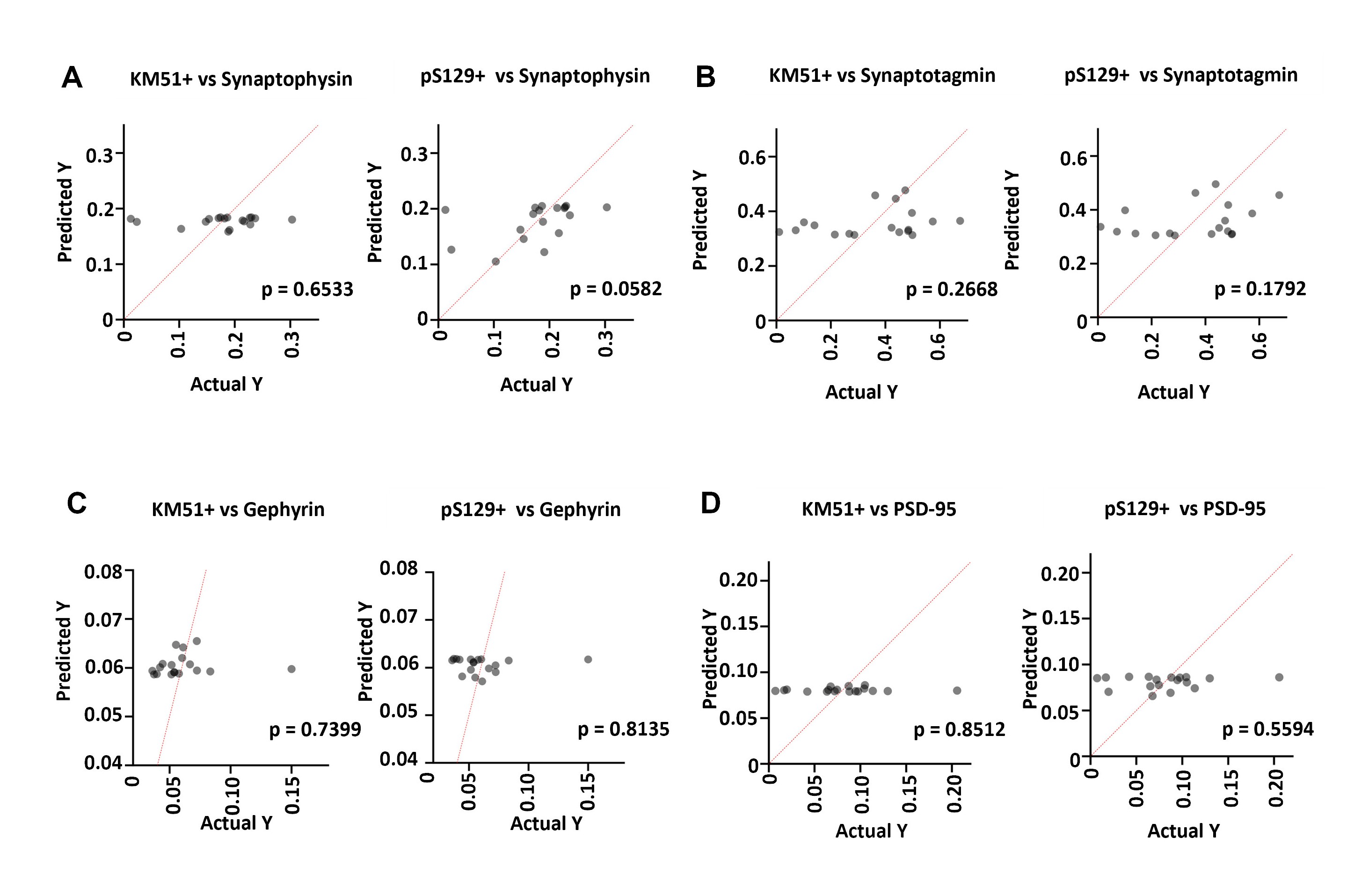

Supplement: Supplementary file 18 — Figure S17: Multiple linear regressions of synaptic proteins against KM51 + and pS129 + LBs in ITG. KM51 + and pS129 + LB, calculated as density per μm. A. KM51 + and pS129 + LB vs. synaptophysin in ITG. B. KM51 + and ps129 + LB vs. synaptotagmin in ITG. C. KM51 + and pS129 + LB vs. gephyrin in ITG. D. KM51 + and pS129 + LB vs. PSD‐95 in ITG. [file NAN-52-e70085-s002.jpg]

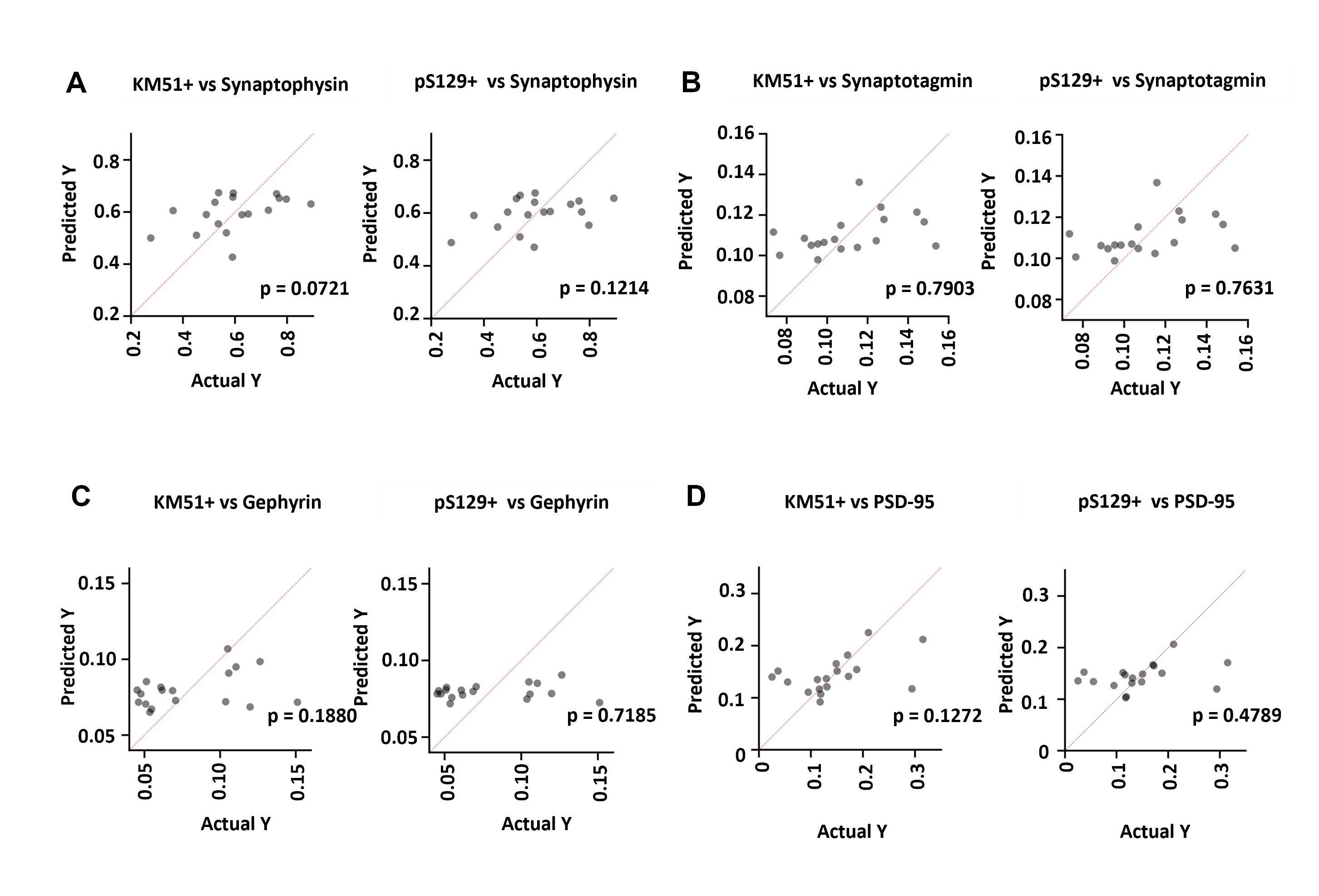

Supplement: Supplementary file 19 — Figure S18: Multiple linear regressions of synaptic proteins against KM51 + and pS129 + LBs, as well as disease duration, in CG. KM51 + and ps129 + LB, calculated as density per μm. A. KM51 + and pS129 + LBs vs. synaptophysin in CG. B. KM51 + and pS129 + LBs vs. synaptotagmin in CG. C. KM51 + and pS129 + LBs vs. gephyrin in CG. D. KM51 + and pS129 + LBs vs. PSD‐95 in CG. [file NAN-52-e70085-s026.jpg]

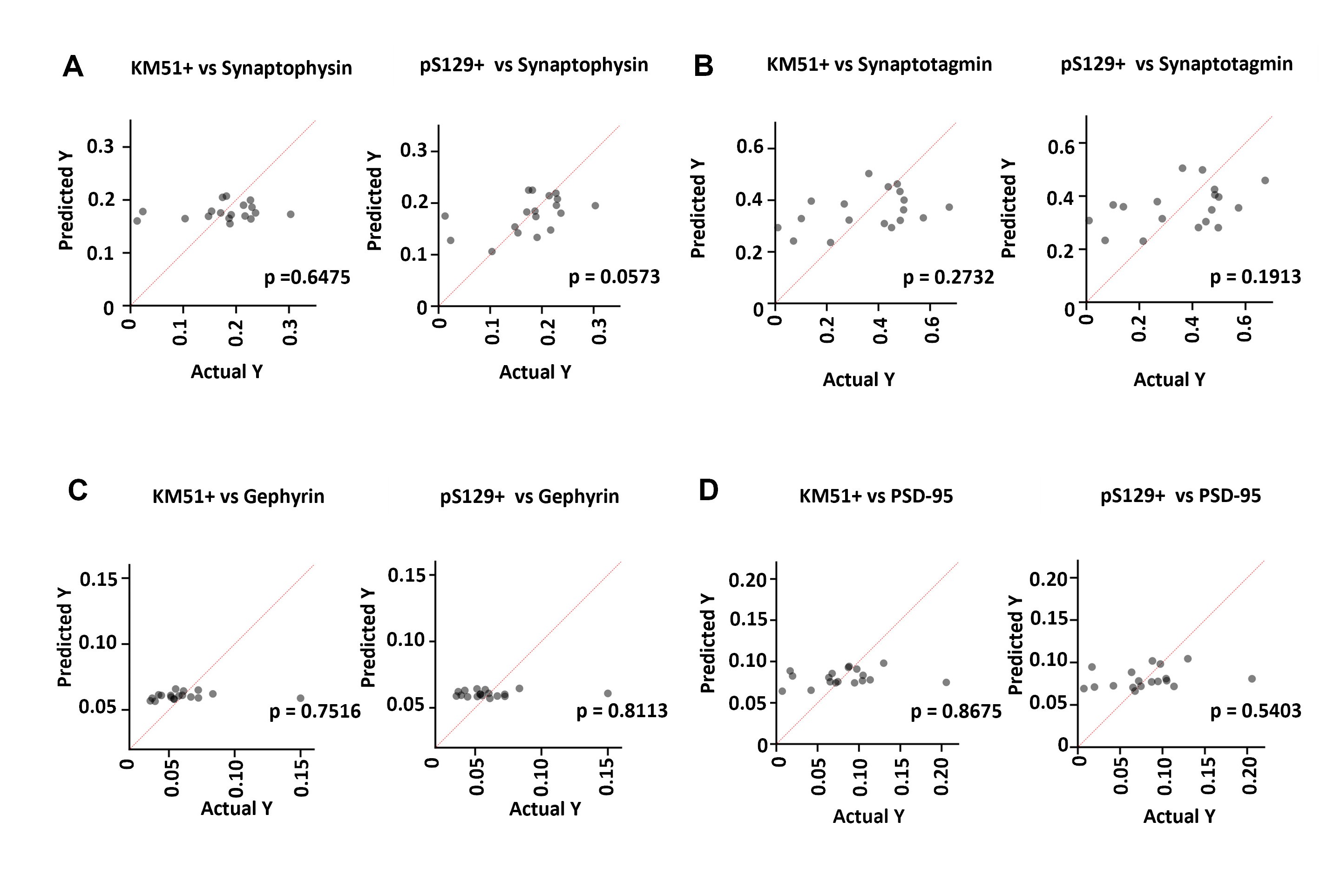

Supplement: Supplementary file 20 — Figure S19: Multiple linear regressions of synaptic proteins against KM51 + and pS129 + LBs, as well as disease duration, in ITG. KM51 + and pS129 + LBs, calculated as density μm. A. KM51 + and pS129 + LBs vs. synaptophysin in ITG. B. KM51 + and pS129 + LBs vs. synaptotagmin in ITG. C. KM51 + and pS129 + LBs vs. gephyrin in ITG. D. KM51 + and pS129 + LBs vs. PSD‐95 in ITG. [file NAN-52-e70085-s017.jpg]

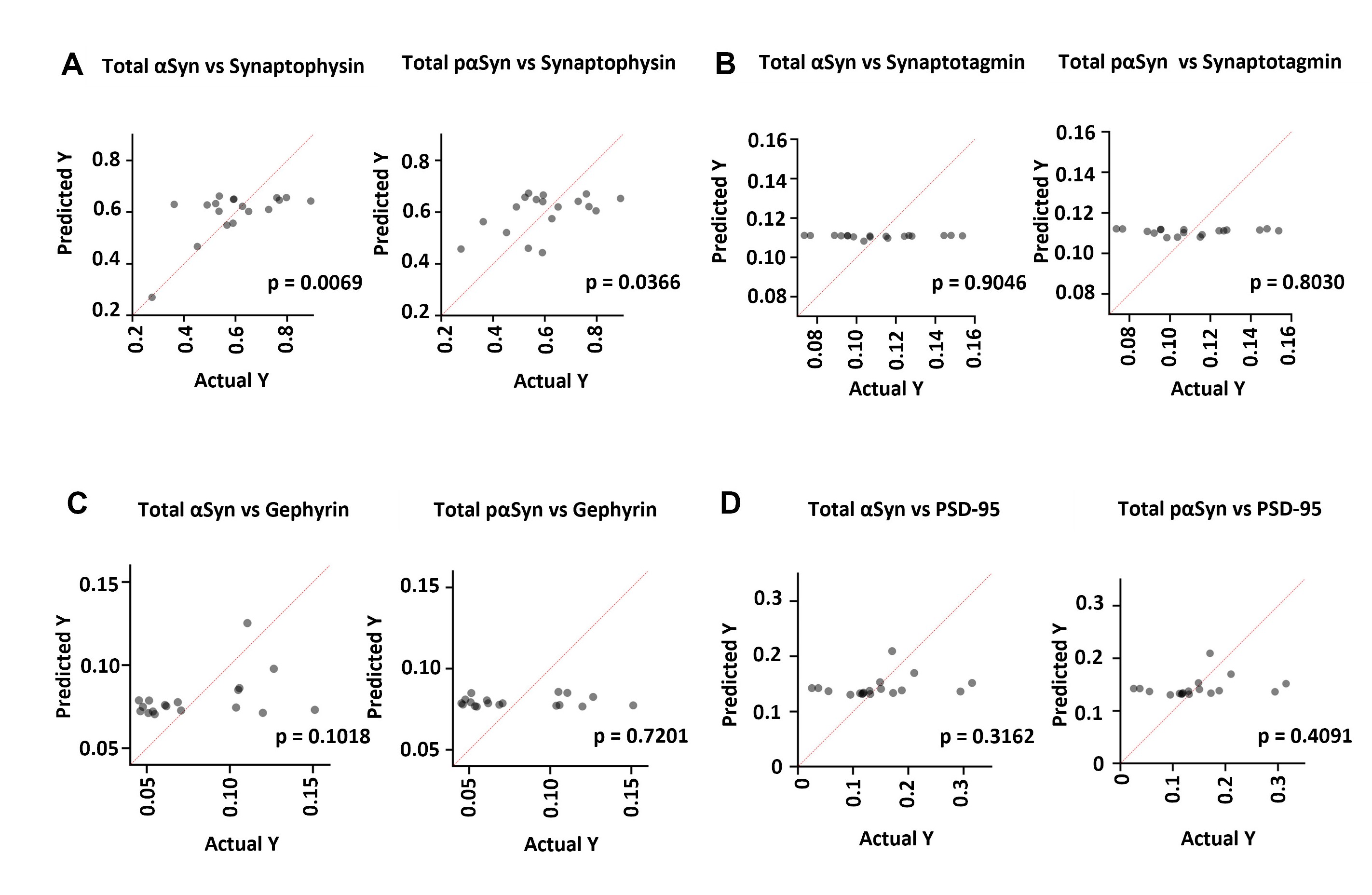

Supplement: Supplementary file 21 — Figure S20: Multiple linear regressions of synaptic proteins against total αSyn and pαSyn by percentage area in the CG. KM51 and ps129 antibodies were used to calculate total αSyn and pαSyn, inclusive of small inclusions such as Lewy neurites. A. Total αSyn and pαSyn vs. synaptophysin in CG. B. Total αSyn and pαSyn vs. synaptotagmin in CG. C. Total αSyn and pαSyn vs. gephyrin in CG. D. Total αSyn and pαSyn vs. PSD‐95 in CG. [file NAN-52-e70085-s013.jpg]

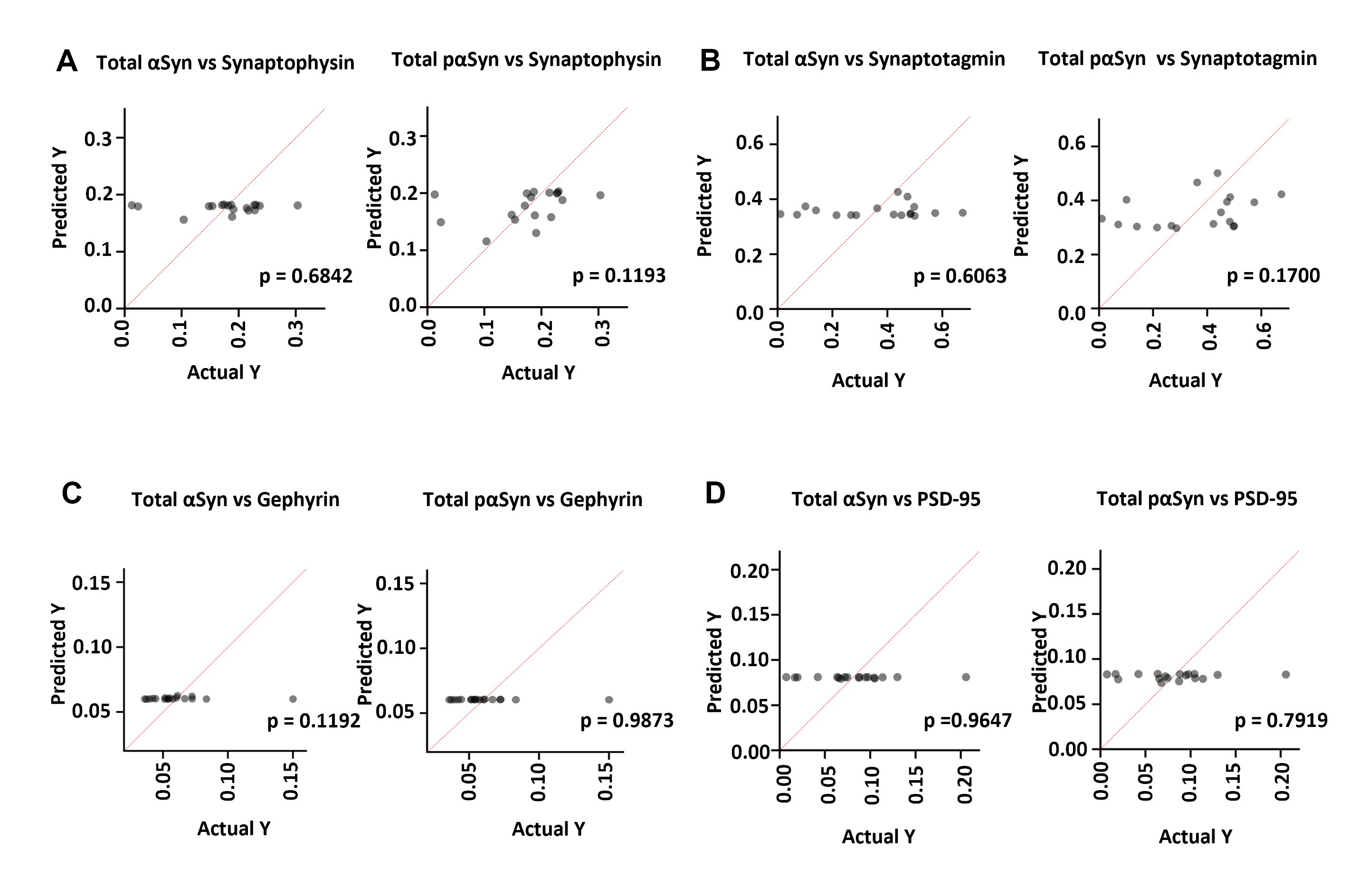

Supplement: Supplementary file 22 — Figure S21: Multiple linear regressions of synaptic proteins against total αSyn and pαSyn by percentage area in the ITG. KM51 and ps129 antibodies were used to calculate total αSyn and pαSyn, inclusive of small inclusions such as Lewy neurites. A. Total αSyn and pαSyn vs. synaptophysin in CG. B. Total αSyn and pαSyn vs. synaptotagmin in CG. C. Total αSyn and pαSyn vs. gephyrin in CG. D. Total αSyn and pαSyn vs. PSD‐95 in CG. [file NAN-52-e70085-s015.jpg]

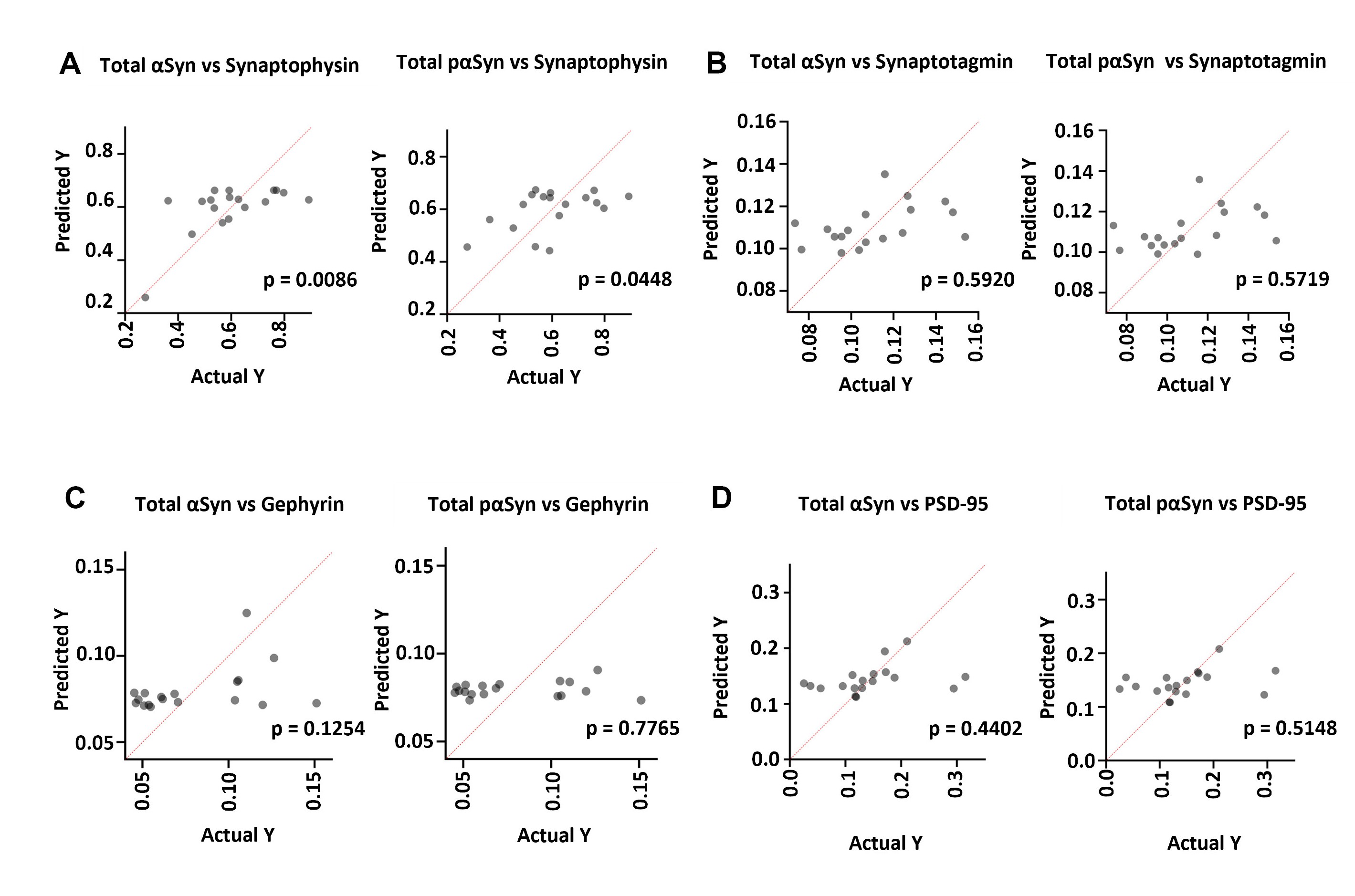

Supplement: Supplementary file 23 — Figure S22: Multiple linear regressions of synaptic proteins against total αSyn and pαSyn by percentage area in the CG with disease duration as a covariate. KM51 and ps129 antibodies were used to calculate total αSyn and pαSyn, inclusive of small inclusions such as Lewy neurites. A. Total αSyn and pαSyn vs. synaptophysin in CG. B. Total αSyn and pαSyn vs. synaptotagmin in CG. C. Total αSyn and pαSyn vs. gephyrin in CG. D. Total αSyn and pαSyn vs. PSD‐95 in CG. [file NAN-52-e70085-s010.jpg]

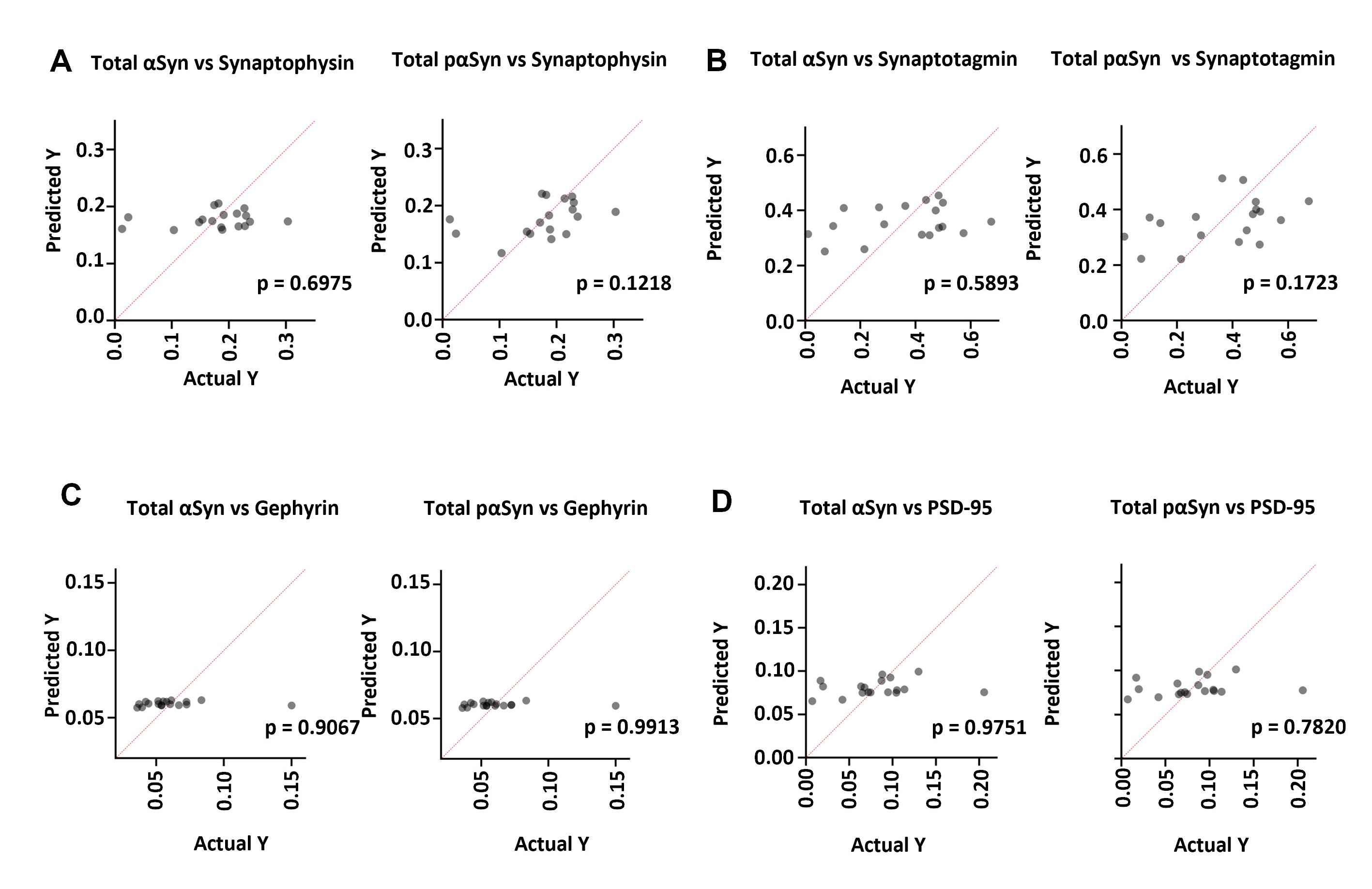

Supplement: Supplementary file 24 — Figure S23: Multiple linear regressions of synaptic proteins against total αSyn and pαSyn by percentage area in the ITG with disease duration as a covariate. KM51 and pS129 antibodies were used to calculate total αSyn and pαSyn, inclusive of small inclusions such as Lewy neurites. A. Total αSyn and pαSyn vs. synaptophysin in CG. B. Total αSyn and pαSyn vs. synaptotagmin in CG. C. Total αSyn and pαSyn vs. gephyrin in CG. D. Total αSyn and pαSyn vs. PSD‐95 in CG. [file NAN-52-e70085-s021.jpg]
